# Socio-economic dynamics of Magdalenian hunter-gatherers: Functional perspective

**DOI:** 10.1371/journal.pone.0274819

**Published:** 2022-10-05

**Authors:** Eugénie Gauvrit Roux

**Affiliations:** 1 ArchaeoZOOlogy in Siberia and Central Asia, CNRS ZOOSCAN IRL 2013, International Research Laboratory, Novosibirsk, Russia; 2 Université Côte d’Azur, CNRS UMR 7264 CEPAM, Nice, France; University at Buffalo - The State University of New York, UNITED STATES

## Abstract

The beginning of the Middle Magdalenian is marked by an increase in the density and geographic extension of evidences of human occupation across western Europe. The Early Middle Magdalenian (19,5–17,5 ka cal. BP) thereby extends from Poland to Spain, and the sharing of the flint-knapping concepts and the circulation of raw materials show the existence of networks active over this wide area. In parallel, part of the production of art, ornaments, microliths, bone industry, and the proportions of hunted ungulates vary regionally and allow to identify distinct technical traditions. Departing from a palethnographic approach at a regional scale, this paper aims at participating in renewing our understanding of the mechanisms of regionalisation during the period, and among past societies of hunter-gatherers. The reflection is based on the techno-functional analysis of stone tools from two cave sites of west-central France that are at the heart of the definition of two technical traditions: La Marche (Magdalenian with Lussac-Angles points) and the Blanchard cave (Magdalenian with navettes). Inter-site comparisons of the functioning and management of stone tools, and of subsistence strategies show the sharing of techno-economical norms, expressing the adhesion to a wider community of practice. The long-term occupation of at least part of the caves and the high density of sites in the Vienne, the Creuse, the Gartempe, and the Charente Valleys, indicate the strong regional implantation of human societies. This strong territoriality (effective and symbolic) is likely a major factor to understand the specificity of the EMM expressions in the area, as well as the sharing, in the same economic territory, of technical norms and of part of the system of symbolic representation.

## 1. Introduction

The beginning of the Middle Magdalenian is marked by an increase in the density and geographic extension of the evidences of human occupation across western Europe. The archaeological sites attributed to the Early Middle Magdalenian (EMM, 19,5–17,5 ka cal. BP) [1–3] are known from Poland to Spain. The EMM develops during the first cold and humid phase of the Heinrich 1 event (H1) [4], and during this period, flint and shells circulated over long distances, as well as the blade and bladelet *débitage* concepts [1, 3, 5–8]. The similarity of part of the lithic industries attests of a unity over this wide area allowed by the existence of tight networks of circulation of ideas, people, or goods, that were active on a wide scale. In parallel, part of the production of art, ornaments, microliths, bone industry, and the proportions of hunted ungulates vary regionally.

West-central France plays a specific role in the expression of the EMM variability. The regional historiography and the richness of art, ornaments, lithic, and osseous material have allowed to recognise early two Magdalenian traditions in west-central France, which were at least partially contemporaneous during the first phase of the H1 event: the Magdalenian with Lussac-Angles points (MLA), first identified at La Marche and the Roc-aux-Sorciers in eastern Vienne [9], and the Magdalenian with Navettes (MN), characterised in particular from the assemblage of the Blanchard cave and the Grand Abri at La Garenne in Indre [10]. The MLA of west-central France is mostly defined by the eponymous short single-bevelled osseous projectile points, the engraving of human realistic profiles on limestone, ivory beads named stomach beads, perforated and notched hyoid bones, and engraved horse incisors. The MN owes its name to the navettes, which are antler bifid artefacts, often found associated with long double-bevelled osseous projectile points, schematised human figures, and phalliform pieces [3, 6, 8–18]. In spite of differences in systems of symbolic representation and weaponry, the MLA and the MN shared an economic territory and both produced truncated backed pieces, which are abundant in the sites of the region and rare elsewhere [1, 3, 6, 19].

Based on a palethnographic approach at a regional scale, this article aims at participating in renewing our understanding of the mechanisms of regionalisation of the expressions of the EMM. The reflection is based on the techno-functional analysis of the stone tools from two cave sites of west-central France that are at the heart of the definition of two technical traditions: La Marche (MLA) and the Blanchard cave (MN). This paper examines and compares, on the intra- and inter-site scales, the functioning and management of stone tools, the subsistence strategies, and the function of the sites. These elements, combined with multiproxy regional data from faunal remains, art, ornaments, and raw material circulation, allow to reconsider the techno-economical dynamics at the EMM in west-central France, and transform our way to conceive the EMM traditions.

## 2. Regional setting

**Fig 1:**
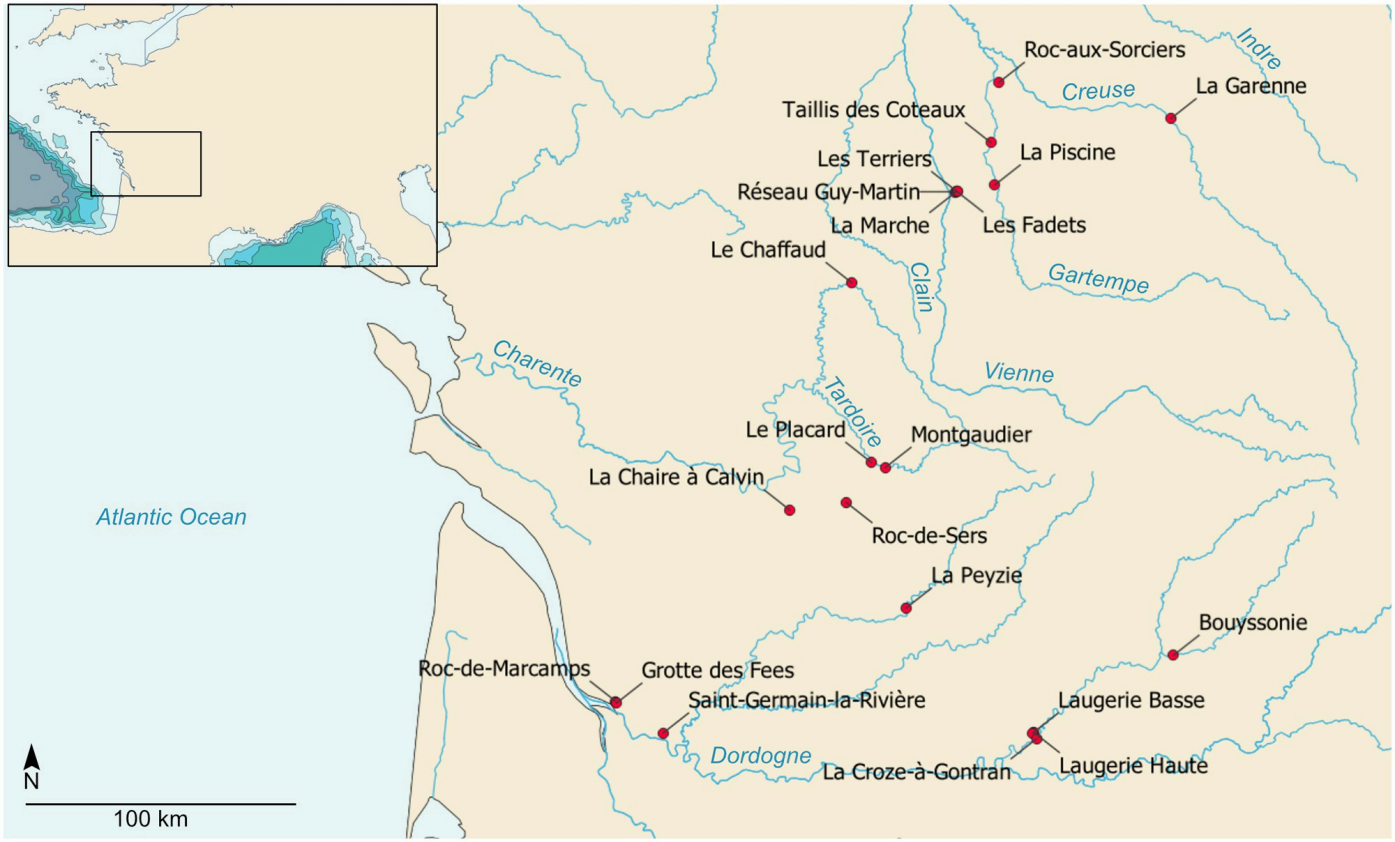
Early Middle Magdalenian cave or rock shelter sites of west-central France. Basemap: Natural Earth, QGis software.

West-central France is composed of wide and open valleys with low elevation reliefs, and of diverse karstic networks that were occupied by prehistoric societies. The region yields a series of EMM cave or rock shelter sites located in the valleys of the Vienne (La Marche, the Réseau Guy-Martin, the Fadets, the Terriers), the Gartempe (the Taillis-des-Coteaux, the Piscine, and the Roc-aux-Sorciers, which includes the Bourdois rock shelter and the Taillebourg cave), the Creuse (the caves of La Garenne, which include the Blanchard cave and the Grand Abri), and the Charente (the Puits du Chaffaud, the Paignon rock shelter at Montmorillon, the Placard, the Roc-de-Sers) (Fig 1). The paleoenvironmental data from the marine [4, 20, 21], glacial [22], and regional continental records [23–28] indicate that the period was marked by cold and relatively wet conditions, and by a landscape dominated by taxa of the cold steppe. Major hunted fauna include ungulates of cold open landscapes such as reindeer (*Rangifer tarandus*), horse (*Equus caballus*), bison and aurochs (*Bovinae*), and saiga antelope (*Saiga tatarica*) [25, 28–30]. Major plant taxa include grasses (*Poaceae*), mugworts (*Artemisia*), asters (*Asteraceae*) and heaths (*Ericaceae*). As during the Last Glacial Maximum, tree taxa–comprising pine (*Pinus*), juniper (*Juniperus*), birch (*Betula*), willow (*Salix*), and oak (*Quercus*)–were typically rare in western Europe and the landscapes were open [10, 21, 23, 26]. The anthracological data are scarce for the EMM and the flora associations remain mostly known through palynological data. Ongoing anthracological analysis at the Bouyssonie cave in Corrèze should bring new elements to discuss paleoecology in western France during the Late Glacial [31].

The EMM traditions of west-central France are mostly defined by the art, ornaments, and osseous productions, which are particularly abundant (e.g., hundreds of engraved limestone slabs at La Marche) or imposing (e.g., monumental sculpted frieze of the Roc-aux-Sorciers). Most research focused on these productions, leading to a reduced interest in the lithic industry [32], which is besides widely considered as part of a common background of the Middle Magdalenian.

**Fig 2:**
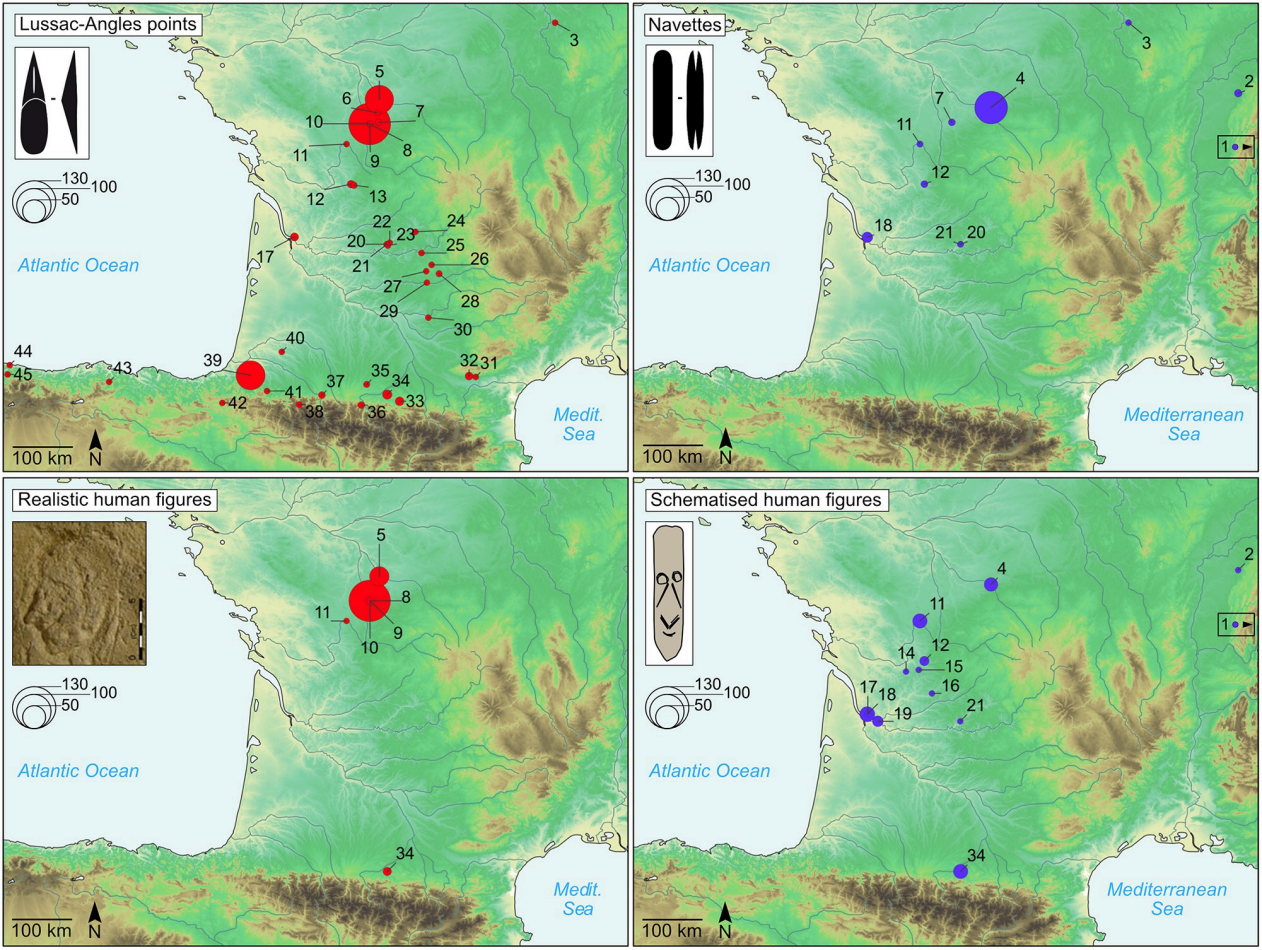
Distribution of the elements characterising the tradition with Lussac-Angles points (red) and the tradition with navettes (blue). 1-Mascycka. 2-The Grappin cave. 3-Trilobite. 4-La Garenne (Blanchard cave, Grand Abri). 5-Roc-aux-Sorciers (Taillebourg cave, Bourdois rock shelter). 6-Taillis-des-Coteaux. 7-La Piscine. 8-La Marche. 9-Réseau Guy-Martin. 10-Les Fadets. 11-Le Chaffaud. 12-Le Placard. 13-Montgaudier. 14-Chaire-à-Calvin. 15-Roc-de-Sers. 16-La Peyzie. 17-The Fées cave. 18-Roc-de-Marcamps. 19-Saint-Germain-la-Rivière. 20-Laugerie-Haute. 21-Laugerie-Basse. 22-La Madeleine. 23-La Croze-à-Gontran. 24-Esclauzure. 25-Combe-Cuiller. 26-The Roussignol cave. 27-Pégourié. 28-Sainte-Eulalie. 29-Les Cambous. 30-Plantade. 31-The Gazel cave. 32-Canecaude I. 33-Enlène. 34-Marsoulas. 35-Les Sciles. 36-The Moulin cave. 37-Les Espélugues. 38-Espalungue. 39-Isturitz. 40-Brassempouy. 41-Harregi. 42-Abauntz. 43-El Mirón. 44-Tito Bustillo. 45-La Güelga. Credits for the picture of realistic human figure from the Roc-aux-Sorciers: O. Fuentes, MC-CNP. Basemap: Natural Earth, QGis software.

The geographical distribution of the art, ornaments, and osseous productions characterising the MLA and the MN shows their high concentration in west-central France, in particular at La Marche and the Roc-aux-Sorciers, and at the Blanchard cave and the Grand Abri at La Garenne, as shown on Fig 2 [2, 3, 16, 33, 34]. It also reveals that the influence of these two traditions extends from Spain to Poland, and is centered on west-central France, south-western France, and north-western Spain:

### 2.1. The Magdalenian with Lussac-Angles points

**Fig 3:**
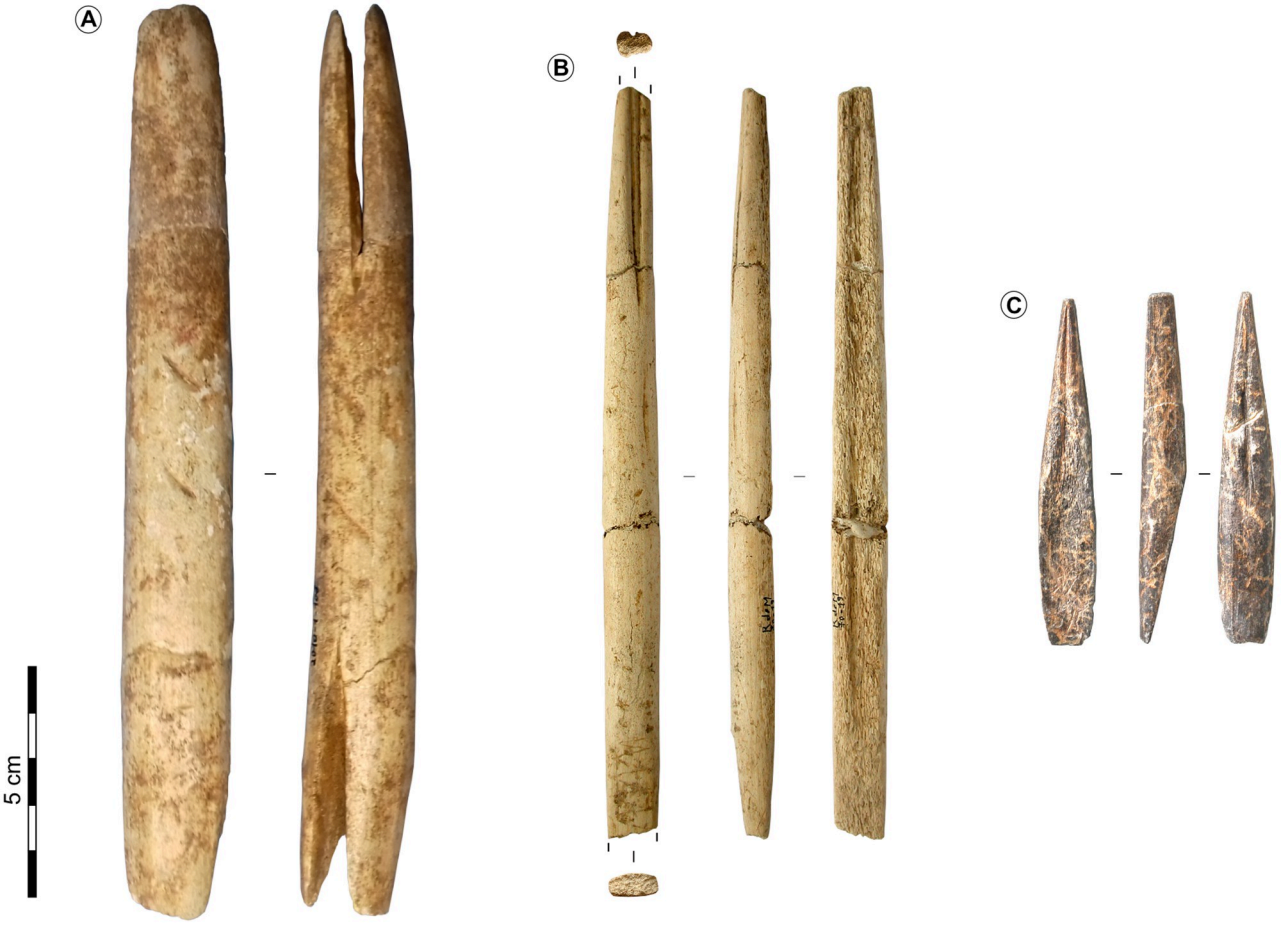
Typical osseous artefacts of the tradition with navettes (A, B) and with Lussac-Angles (C). A-Navette from the Blanchard cave n°2010.1.106, layer B4, credits: A. Dessolier, Musée d’Argentomagus. B-Double-bevelled point from the Roc-de-Marcamps, Maziaud collection, credits: Pétillon, 2016. C-Lussac-Angles point from La Marche, credits: J. Airvaux.

The MLA is mainly defined by the eponymous short, single-bevelled, grooved antler projectile points that have a lanceolate shape and are found from the Yonne department in France to northern Spain (Figs 2 and 3) [2, 3, 9, 34]. La Marche and the neighbouring Roc-aux-Sorciers are the reference sites of the MLA. These sites are about 40 km apart and provide more than half of the known European specimens of Lussac-Angles points. Except from a few Pyrenean sites, these points specimens are generally found isolated outside of the east of the Vienne department (Fig 2) [2, 34, 35].

The engraving or sculpting of realistic human faces and bodies on cave walls or slabs also plays an essential part in the regional expression of the EMM (Fig 2) [15–17, 36, 37]. This theme is most abundant at La Marche which yields 110 engravings of human figures [37] and at the Roc-aux-Sorciers, which yields 21 engraved figures [16]. Though anecdotal, the theme is also found in several other EMM sites: the Fadets, the Réseau Guy-Martin, the Chaffaud, and, 400 km away, in Marsoulas. The art production is furthermore rich with numerous animal representations which were sculpted and painted or engraved. The diversified bestiary notably includes bison, ibex, horses, and reindeers [14, 15, 38, 39].

The horse incisors engraved with geometrical patterns often accompany the Lussac-Angles points in west-central France. La Marche yields the largest quantity, with about 100 engraved teeth; the Roc-aux-Sorciers yields nearly 30 pieces, and isolated specimens are found in Vienne and Charentes sites including the Fadets, the Chaffaud, Montgaudier [3, 40–44]. Other ornament productions are found in association with the Lussac-Angles points: the ivory beads (or stomach beads) are specific to the sites of eastern Vienne, and the pierced and notched hyoid bones are found from the Saône-et-Loire in France to the Asturias in Spain [8, 15].

### 2.2. The Magdalenian with navettes

The Blanchard cave is a reference site of the Magdalenian with navettes, which are antler bifid pieces often interpreted as tools hafts, and named by analogy with shuttles of textile workers (Figs 2 and 3) [10, 17, 34, 45]. They are found from Poland to the Gironde department in France. The Grand Abri and the Blanchard cave on the hillside of La Garenne yield over half of the European specimens, with nearly 90 fragmented or whole navettes [3, 10, 33, 39, 46].

Long double-bevelled grooved osseous points with a quadrangular section are often associated with the navettes assemblages. Their geographical distribution is similar to the navettes, and the caves of La Garenne also yield most of the European specimens [2, 3, 10, 46] (Figs 2 and 3).

The engravings of schematic human representations on osseous material are associated with the navettes in several sites. These representations are found in 12 sites from the Jura to the Haute-Garonne departments, where they are always few, with 10 engraved pieces or less per site (Fig 2) [3, 10, 15, 16, 46, 47]. Contrary to the MLA, the art of the MN is schematic or abstract, it is rarely on mineral but rather on osseous materials, notably reindeer antler; the blanks can be osseous artefacts such as chisels or navettes [16, 48, 49]. The phalliform pieces are frequently associated with the navettes, the double-bevelled points and the schematic human faces. They are partial schematised human representations frequently realised on perforated batons made of reindeer antler. They are found from the Doubs to the Pyrénées-Atlantiques departments and are generally isolated in the sites; La Garenne and the Roc-de-Marcamps are the only sites yielding about 10 specimens each [3, 10, 46].

## 3. The corpus of sites

The concentration of the classic markers of the MLA and the MN at La Marche and the Blanchard cave confers to these sites a privileged role to understand the mechanisms of diversification of the EMM expressions. The sites are 60 km away and set in similar environments: they are small limestone caves exposed to the south and dominate a watercourse from a low hillside.

The Blanchard cave was discovered in 1956 and excavated until 1976 [50, 51], evidencing a stratified site whose layers are all associated with the EMM. It is composed of six main layers (B2, B3, B4, B4+B5, B5, and B6), which all yield navettes [10, 52]. The Bayesian modelling of the ages of the earliest layers of the Blanchard cave (B4 to B6) dates their occupation from circa 18,6 to 18,1 cal. ka BP [53]; the youngest layers B3 and B2 are not dated yet, but are attributed to the EMM by relative dating.

La Marche was excavated discontinuously from 1937 to 1957 [40, 54], and the excavated material was screened between 1988 and 1993 [55]. The site yields one single and rich EMM layer. It is composed of a palimpsest of occupations, and its thickness varies between 15 and 30 cm. The Bayesian modelling of the dates of this layer shows that the occupations occurred between circa 18,2 and 17,3 cal. ka BP [56–58].

The faunal assemblages of both sites are particularly rich, with over 10000 remains at La Marche, and 31702 remains counted at the Blanchard cave in the layers B3, B4 (back of the cave), B5 (back of the cave), and B6 [25]. The bones are highly fragmented and, in both sites, the exploited fauna is diversified and focused on the hunt of horses and reindeers. At the Blanchard cave, the secondary taxa include wolf (*Canis lupus*), saiga antelope, hare (*Lepus sp*.), ibex (*Capra ibex*), bison or aurochs, fox (*Vulpes sp*.*/Alopex sp*.), chamois (*Rupicapra rupicapra*), bear (*Ursus arctos*), boar (*Sus scrofa*), and mustelids (*Mustelidae*). At La Marche, the secondary taxa include bovines (*Bovidae sp*.), saiga antelope, cave bear (*Ursus spelaeous*), badger (*Meles meles*), red deer (*Cervus elaphus*) and wolf (*Canis lupus*) [54]. The revision of the faunal assemblage of La Marche is ongoing and should bring new elements regarding the acquisition and exploitation of fauna and the taphonomy of the site [59].

The lithic assemblage of the Blanchard cave is composed of 11735 artefacts (counts P. Paillet, unpublished), and La Marche yields over 10000 artefacts (counts J.-M. Leuvrey, unpublished). In both sites the exploited flints mainly come from the allochthonous sources of the Upper Turonian of the Grand-Pressigny region and from the Lower Turonian of the Indre and Cher Valleys. The exploitation of local flints (Bathonian/Bajocian) is marginal. The anecdotic presence of extra-regional flints from the Aquitaine Basin should be noted: grain de mil from Charente-Maritime in the assemblage of La Marche and of the Blanchard cave, and Senonian from Dordogne and Charente in the assemblage of the Blanchard cave (Primault, pers. com.) [60].

## 4. Methods

The analysis of the archaeological material includes two parts: the technological analysis of lithic assemblages, which aims at reconstructing the methods, objectives, and economy of tools production [61–63], and the use-wear analysis, which examines the functioning, design, and economy of stone tools, and past techniques or processes involved in tools use [64, 65]. The combined techno-functional approach applied to the lithic assemblages allows a systemic restitution of technic behaviours and economic objectives, and enables detailed comprehension of prehistoric techno-economy. This method is therefore particularly adapted to address the question of variability of industries, and allows to renew the dynamics of reflection regarding societal trajectories and interrelation between human behaviours and environments.

The methods of the technological analysis were applied to part of the lithic assemblage of each site, including 5568 pieces from La Marche (collection of the Musée Sainte-Croix, Poitiers, France) (S1 Table), and 1406 pieces from layers B3, B4, B5, and B6 of the Blanchard cave (collection of the Musée d’Argentomagus, Saint-Marcel, France) [66, 67] (S2 Table). Previous works complete the analysis of the production process in this last cave [52, 68, 69].

**Fig 4:**
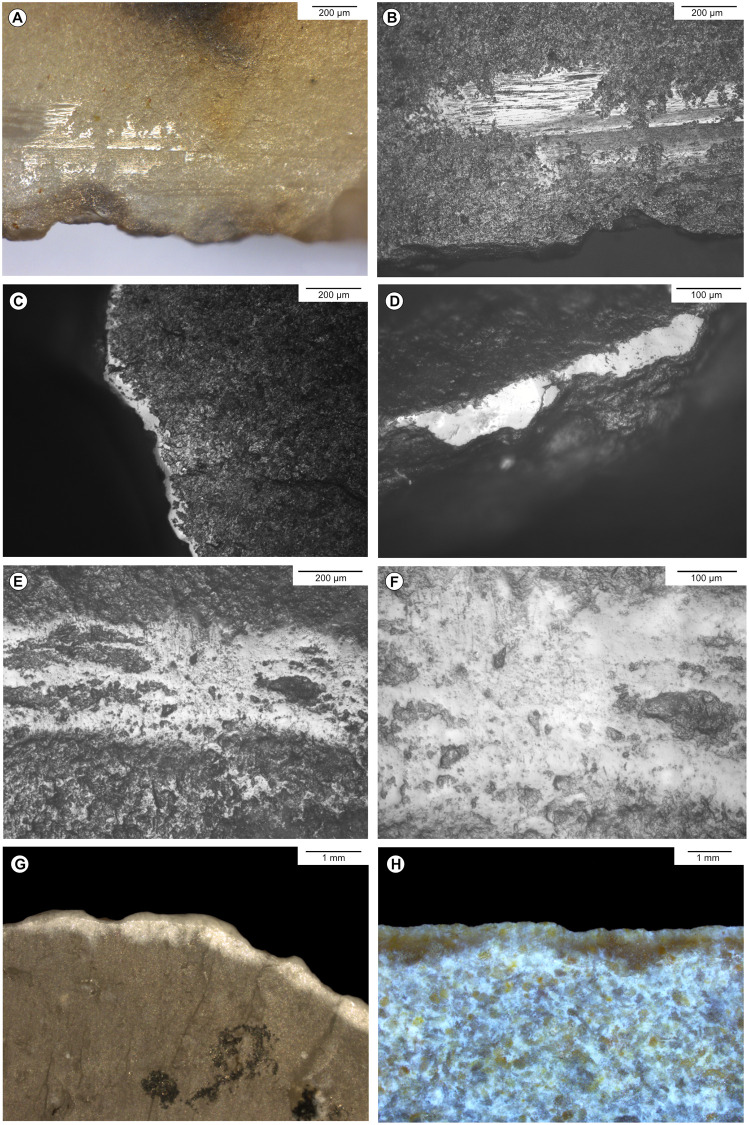
Examples of post-depositional alteration on the archaeological material. A, B-Striated bright spot on a bladelet from the Blanchard cave, layer B5, credits: Gauvrit Roux, 2019. C-Marginal non-striated bright spots on the distal edge of an endscraper from the Blanchard cave, layer B3. D-Marginal non-striated bright spots on the distal edge of an endscraper from the Blanchard cave, layer B4/B5. E, F-Rounding and bright spot on the ridge of a burin from La Marche, credits: Gauvrit Roux, 2019. G-Marginal white patina on the edge of an endscraper from La Marche. H-Fresh scars interrupting white patina on the lateral edge of a burin from La Marche.

The macroscopic sampling for the use-wear analysis aimed at selecting a wide diversity of pieces with potential use damages (fractures, scars, rounding, shine and polish, striations, residues). The selection includes 404 tools from La Marche, and 351 pieces from the Blanchard cave. The sample was analysed using the classic methods of use-wear analysis combining micro- and macroscopic optical observation of the surface of lithic artefacts (from the naked eye to × 200), examining relations between damages due to use, manufacture, and post-depositional alteration (Fig 4). After a preliminary observation with a binocular, the artefacts without residues were softly cleaned with soap under running water, and locally with alcohol to remove sediment, dust, and handling grease from the surfaces.

**Fig 5:**
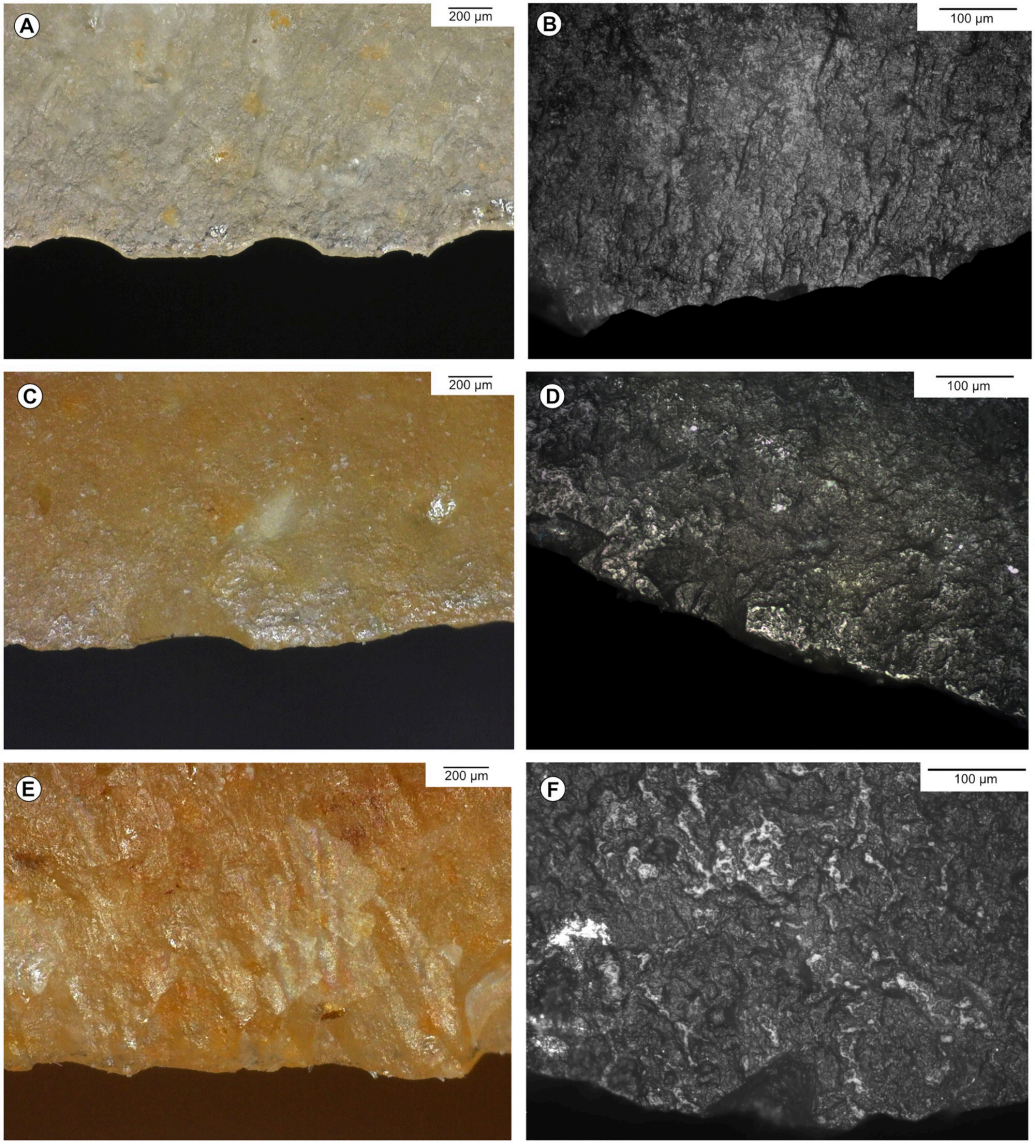
Experimental vegetal working traces on flint. A, B-Cutting nettle, 10 mn. C, D-Sawing fresh chestnut tree, 10 mn. E, F-Sawing fresh lime tree, 10 mn.

**Fig 6:**
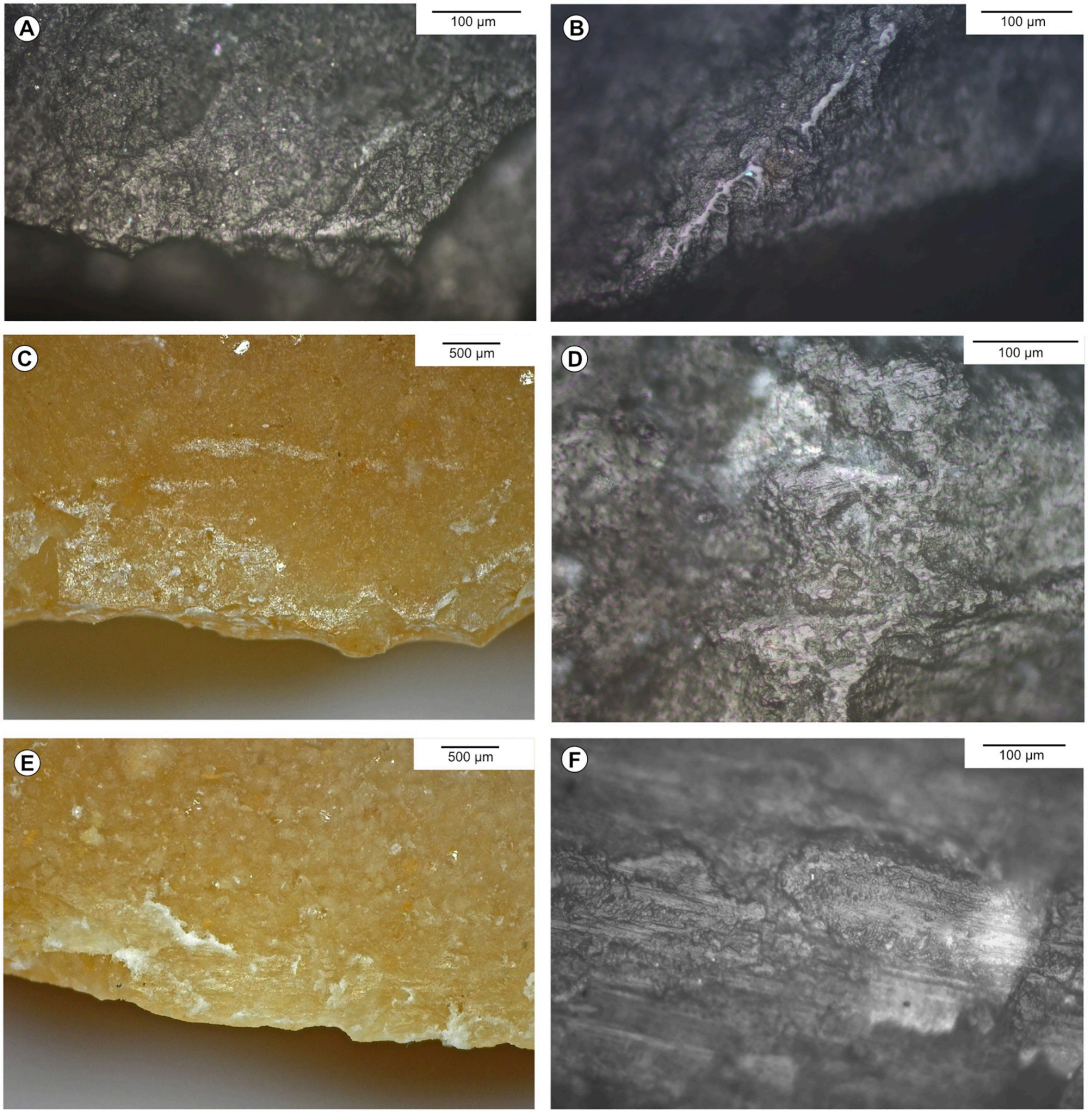
Experimental bone and antler working traces on flint. A-Scraping fresh bone, 180 mn. B-Sawing moisturised antler, 15 mn. C, D-Sawing dry bone, 10 mn. E, F-Sawing dry antler, 10 mn.

**Fig 7:**
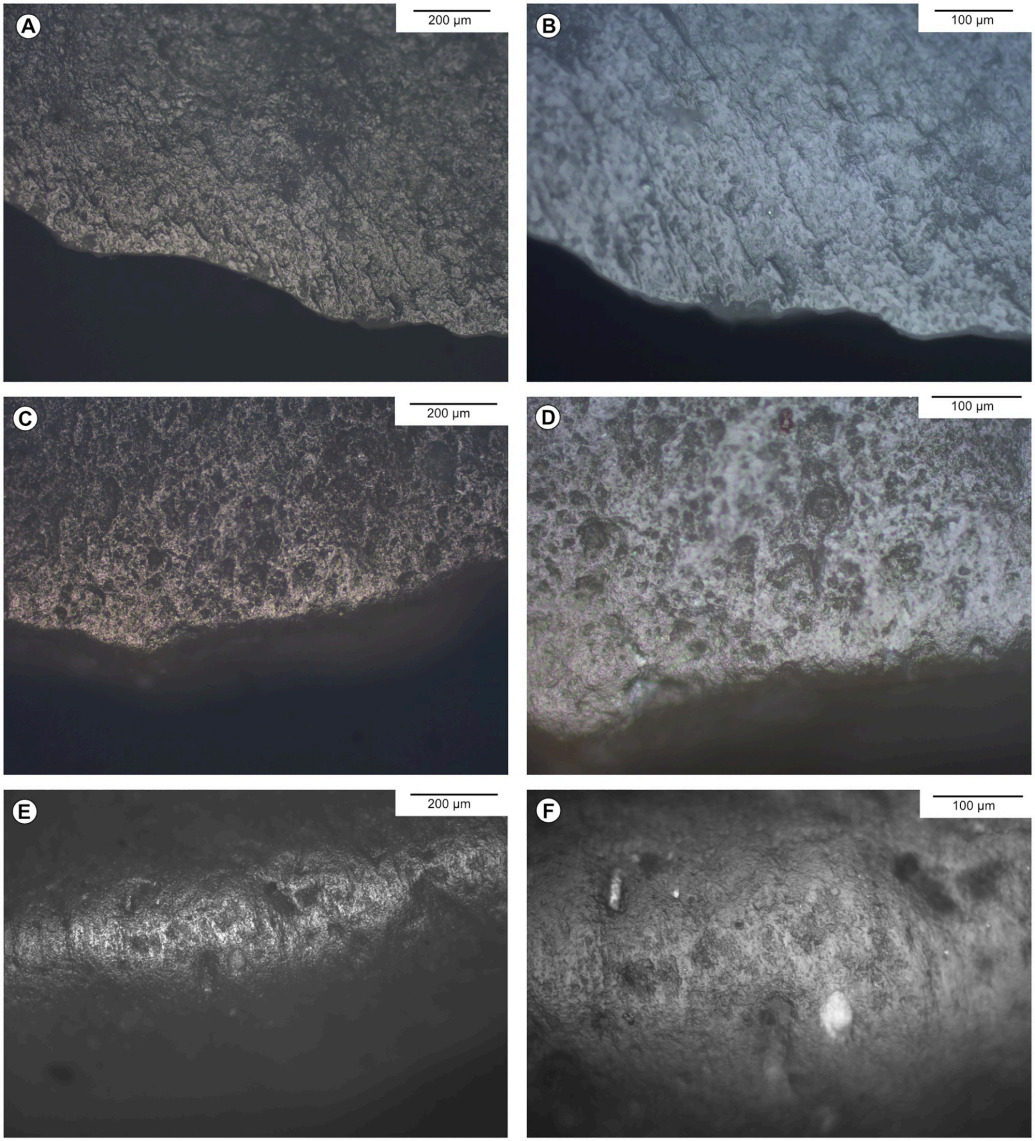
Experimental hide working traces on flint. A, B-Cutting dry hide soaked in water, 30 mn. C, D-Scraping ochred dry hide with punctual moisturising, 30–40 mn. E, F- Scraping dry hide, 30–40 mn.

The observation of the archaeological material was paired with experimentations of production, use, and alteration of backed bladelets [70], experimentations of bison, cow, and roe deer hide working (S. Beyries org., unpublished), and with more specific tests focused on mineral [66], vegetal (Fig 5), shell, bone and teeth working, extraction of reindeer antler using incision, grooving, sawing and percussion (Fig 6), blind-tests, and with the observation of the large experimental reference collection of the CEPAM laboratory in Nice, regarding notably hide working traces (Fig 7).

## 5. Techno-economic role of La Marche and the Blanchard cave

### 5.1. Blades and bladelets production

The *débitage* aimed at obtaining standardised blades and bladelets produced with a soft hammer direct percussion, probably organic: blanks typically have a punctiform butt with an abraded and slightly scarred overhang, a diffuse bulb, an impact point which is rarely visible, a small lip can generally be observed on the lower face, and the flaked surface of cores is slightly careenated [71].

**Fig 8:**
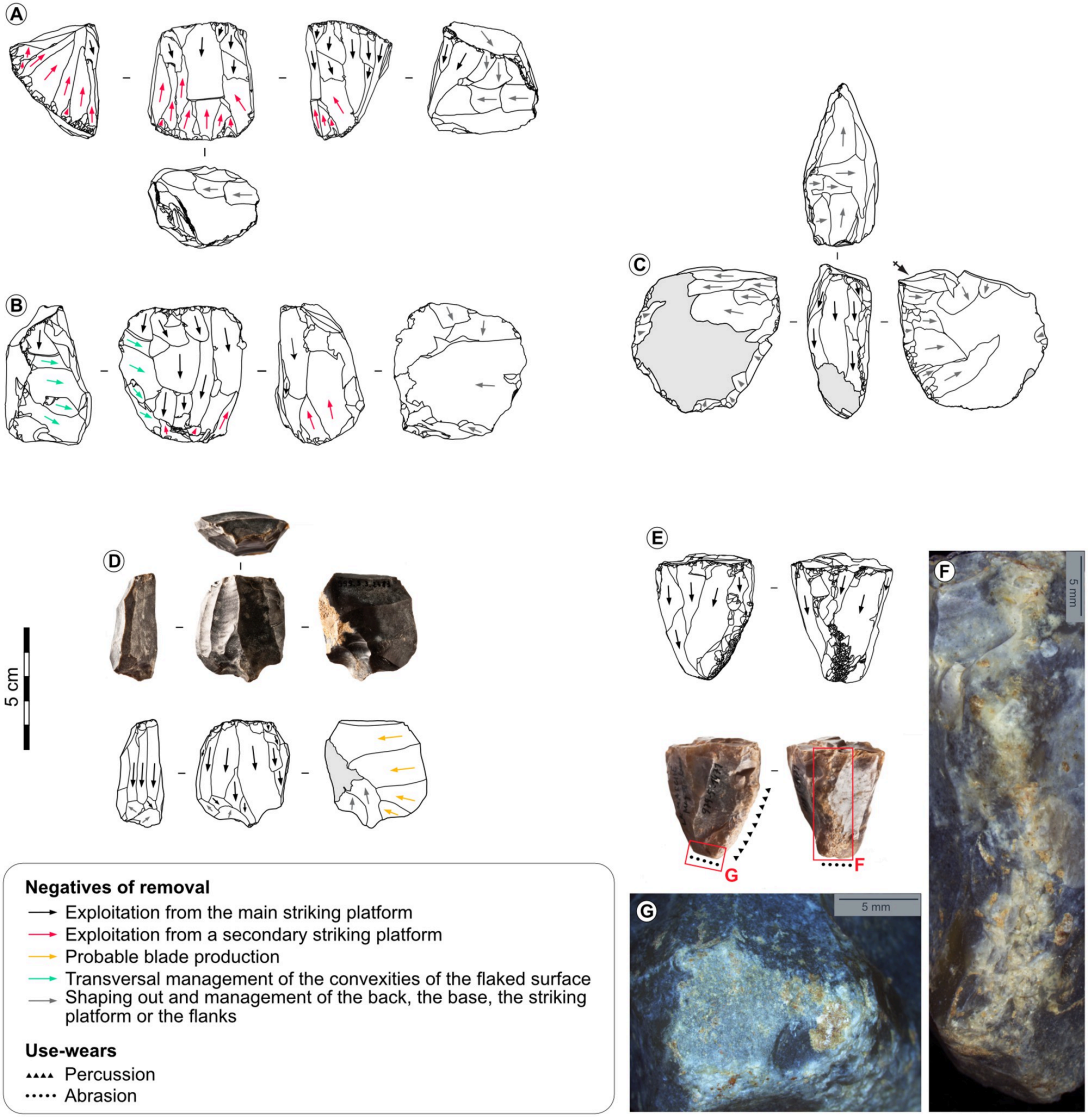
Bladelet cores from La Marche. A, B, D-Prismatic cores on block. C-Core on cortical flake. E-Pyramidal core on block recycled to strike and abrade hard materials. F-Percussion wears on core n°E. G-Abrasion wears on core n°E, credits: Gauvrit Roux, 2019.

Dimensions of smallest blades and largest bladelets overlap (S1 Fig), and the numerous unretouched blanks whose dimensions are intermediary between blades and bladelets might suggest the continuous exploitation of blade cores to produce bladelets. Blades and bladelets however appear to have been mostly produced according to distinct schemes: blades were produced from blocks following a unipolar flaking and a semi-rotating rhythm, and bladelet production was carried out from blocks, flakes, and probably truncated blades at La Marche [19, 72], following a unipolar preferential flaking or a unipolar flaking and a rotating, semi-rotating, frontal, and probably facial structure (Fig 8). Blade and bladelet production therefore appear mostly dissociated. The size limit between retouched blades and retouched bladelets can be set between 10–11 mm width and 4–5 thickness at the Blanchard cave, and between 12–13 mm width and 6–7 mm thickness at La Marche. Bladelets are slightly bigger at La Marche than at the Blanchard cave, possibly due to distinct excavating methods. The retouched bladelets are on average 7.1 mm wide (standard deviation 1.5 mm) and 2.8 mm thick (standard deviation 0.9 mm) at the first site, and 5.1 mm wide (standard deviation 1.2 mm) and 2.3 mm thick (standard deviation 0.7 mm) at the second site. Blades are robust, being on average 25.7 mm wide (standard deviation 7.6 mm) and 8.4 mm thick (standard deviation 3.6 mm) at La Marche, and 21 mm wide (standard deviation 6.2 mm) and 6.4 mm thick (standard deviation 2.6 mm) at the Blanchard cave for the tools without modification of the lateral edge. The blank elongation is not pronounced and blades rarely exceed 80 mm long since most of them are broken (77% at the Blanchard cave and 83% at La Marche), tools are often shaped on one or both extremities, and the successive sharpening aiming at improving efficiency of the working edge during use necessarily caused length reduction of the tool (see below). Blades and bladelets were shaped into distinct tool types intended for different activity spheres. The bladelets were transformed into backed bladelets, very rarely into microperforators, and the equipment on blade is dominated by endscrapers, burins, beaks and perforators, truncated blades, and blades with lateral retouch [66, 67].

No autonomous production of flakes is recognised, and these blanks are only rarely retouched. The production wastes of the bladelet knapping process are numerous in both caves, suggesting that the production occurred at the sites. On the reverse, the scarcity of blade cores, crested blades, and rejuvenation flakes, tends to indicate a segmentation in time and space of the blade production, with the introduction of knapped blanks, or even retouched blades, at the sites.

### 5.2. Use and management of blades

**Fig 9:**
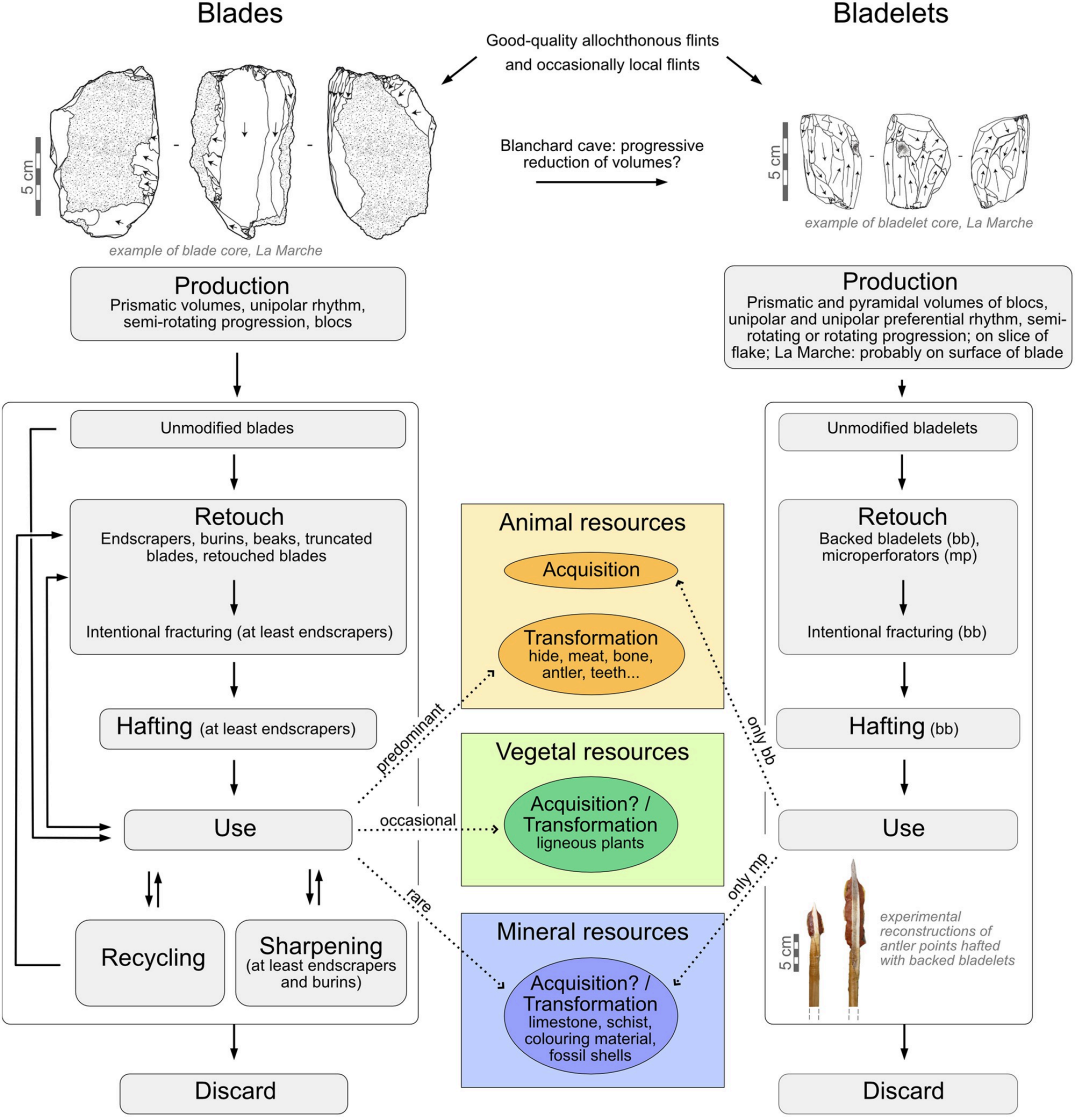
General *chaîne opératoire* from production to discard of blades and bladelets evidenced at La Marche and the Blanchard cave.

**Fig 10:**
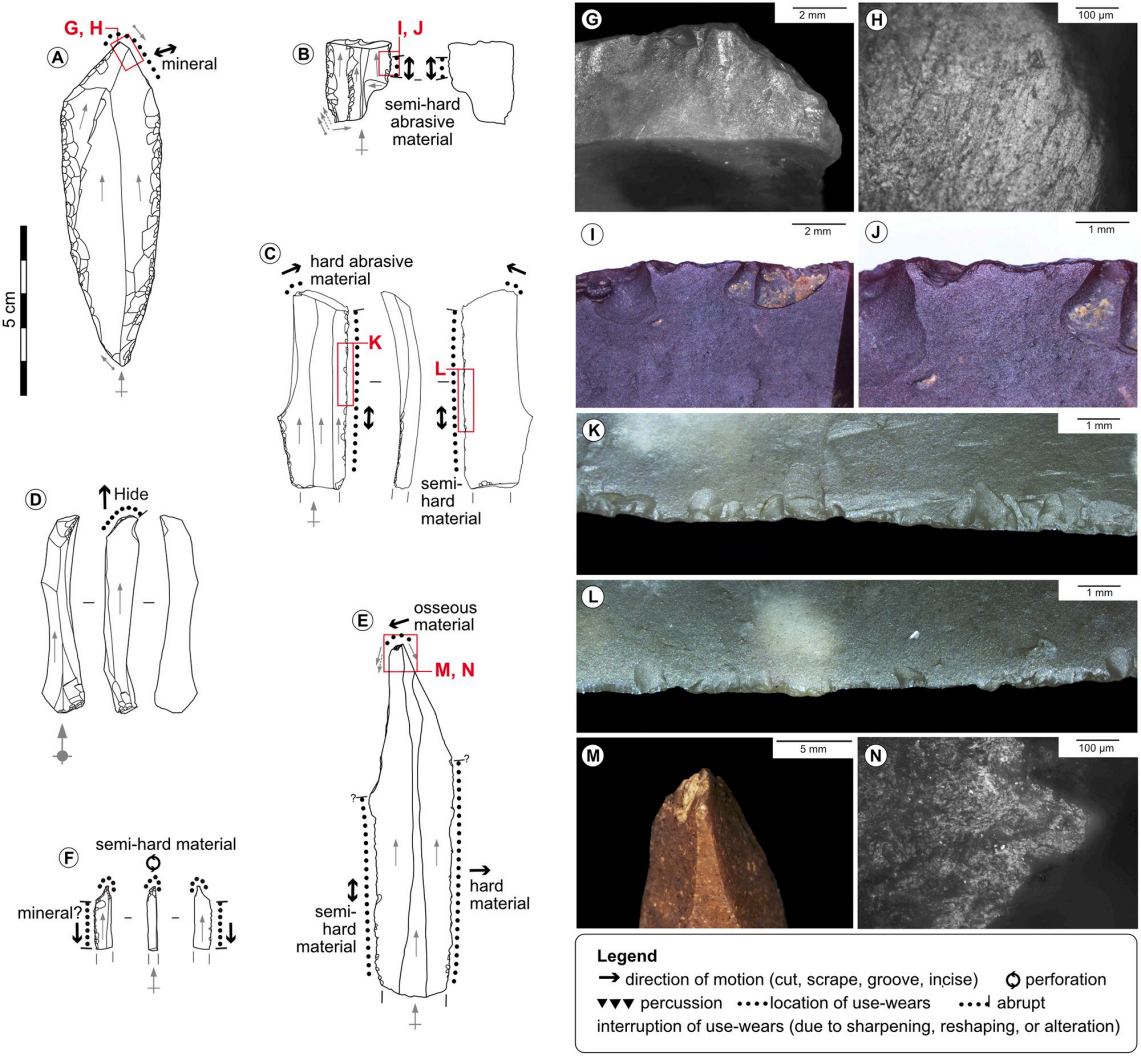
Used burins and spalls. A-Double burin on retouched blade from the Blanchard cave, layer B4. B-Burin on retouched and truncated blade from the Blanchard cave, layer B4. C-Burin on retouched blade from the Blanchard cave, layer B3. D-Large burin spall cutting through a distal endscraper from the Blanchard cave, layer B3. E-Burin on blade from La Marche. F-Microperforator on burin spall from La Marche. G, H-Wears of scraping an abrasive mineral with the distal burin facet of tool n°A, credits: Gauvrit Roux, 2019. I, J-Rounding of sawing a semi-hard abrasive material, interrupted by direct removals on the lateral edge of tool n°B. K, L-Scars due to sawing a semi-hard material with the lateral edge of tool n°C. M, N-Wears of grooving an osseous material with the burin bevel of tool n°E, credits: Gauvrit Roux, 2019.

**Fig 11:**
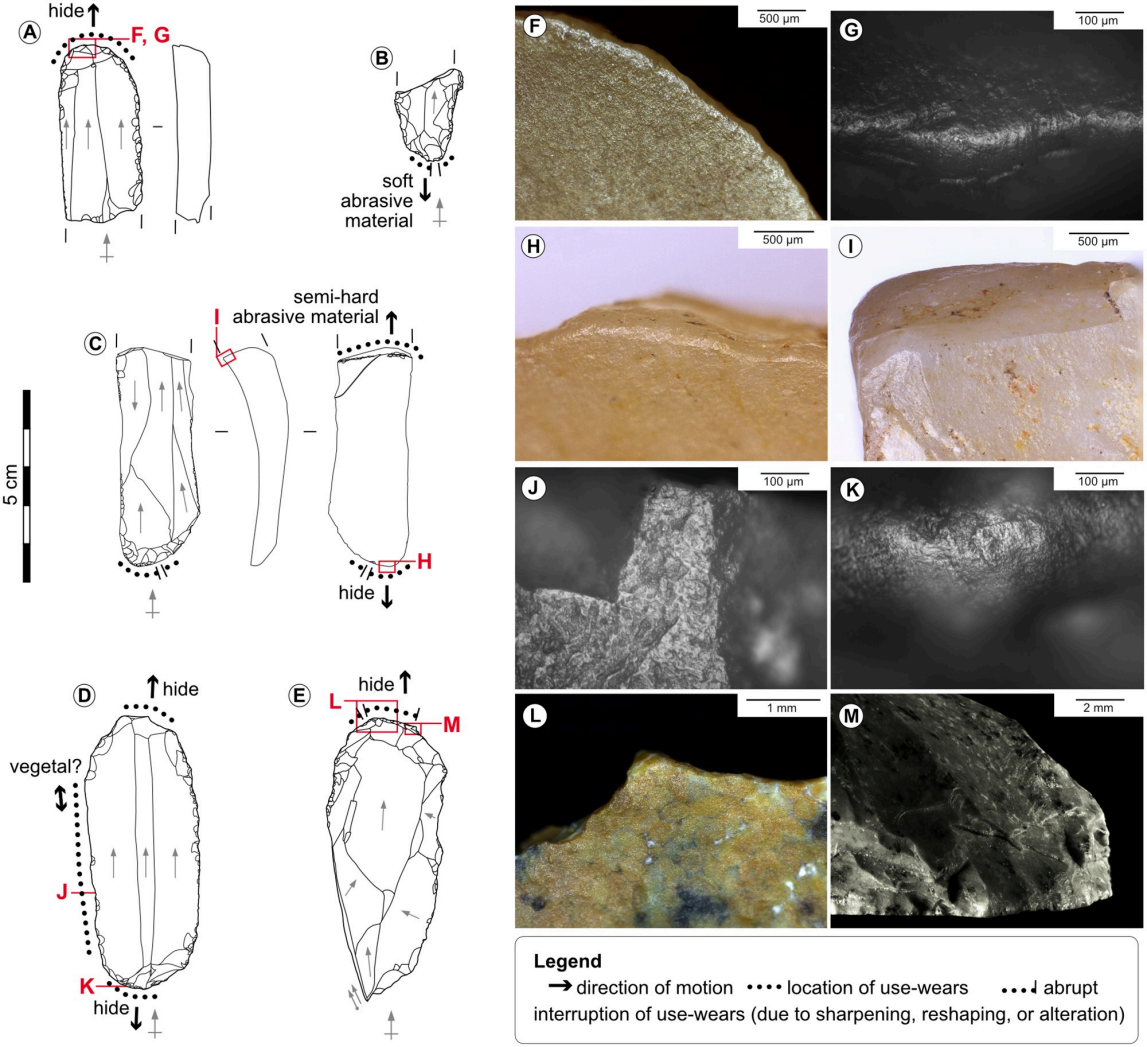
Used endscrapers. A-Endscraper on retouched blade from the Blanchard cave, layer B4. B-Endscraper on retouched blade from the Blanchard cave, layer B4/B5. C-Endscraper on blade from the Blanchard cave, layer B4/B5. D-Double endscraper on blade from La Marche. E-Endscraper-burin from La Marche. F, G-Rounding, shine, striations and polish of dry hide scraping on the distal end of tool n°A, credits: Gauvrit Roux, 2019. H-Rounding and striations due to dry hide scraping on the proximal end of tool n°C. I-Profile view of the fracture edge of tool n°C showing rounding due to scraping of a semi-hard abrasive material. J-Probable wood sawing micro-wears on the lateral edge of tool n°D. K-Dry hide scraping micro-wears on the proximal end of tool n°D. L-Hide rounding interrupted by direct sharpening retouch on the distal end of tool n°E. M-Abrupt edge caused by successive sharpenings of the distal end of tool n°E.

**Fig 12:**
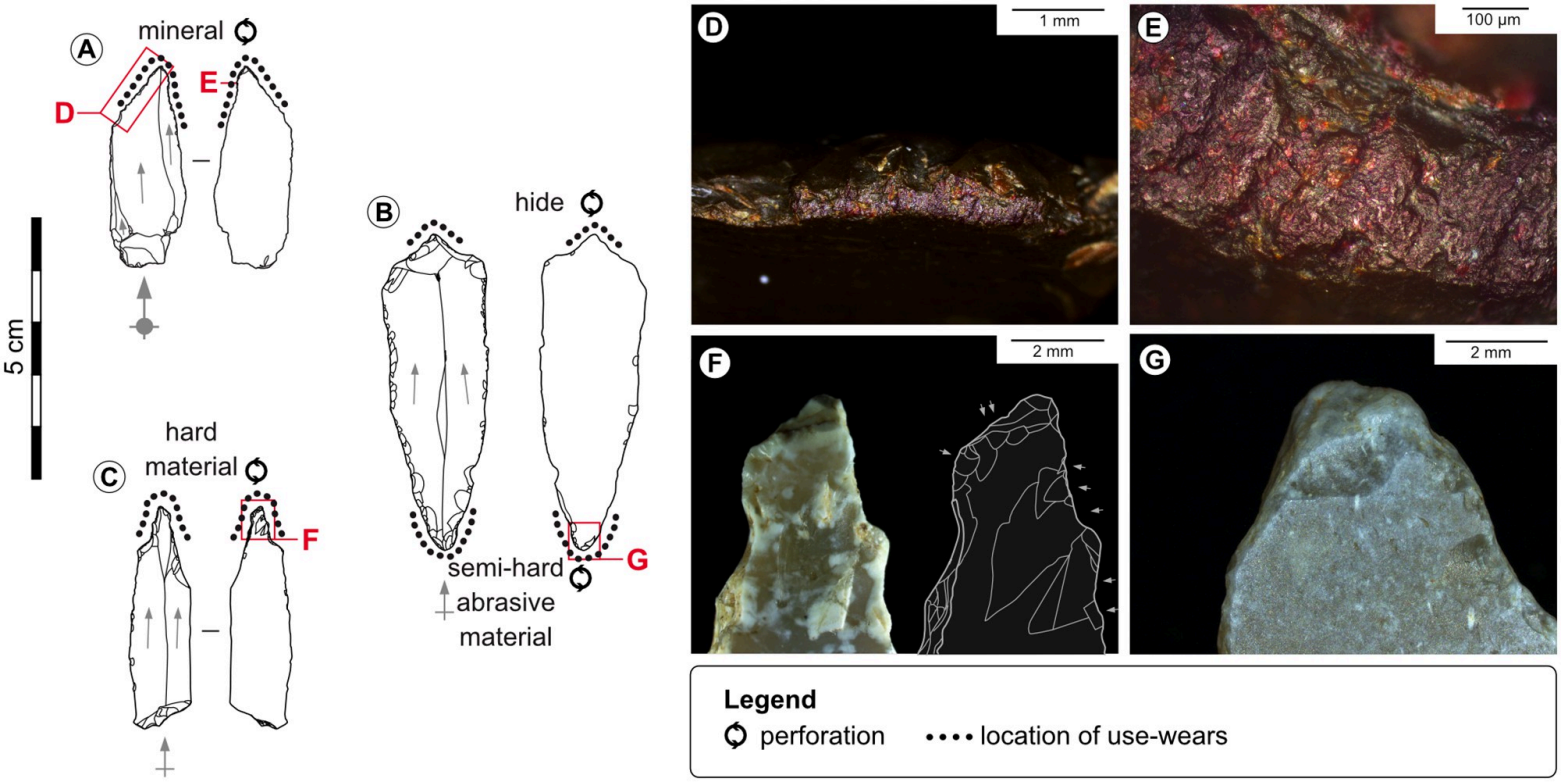
Used beaks and perforators. A-Microperforator from the Blanchard cave, layer B5. B-Double beak from La Marche. C-Microperforator from La Marche. D, E-Wears and residues on the distal point of tool n°A due to perforating a colouring mineral with particularly thick abrasive particles, credits: Gauvrit Roux, 2019. F-Scars due to perforating a hard material with the tip of tool n°C. G-Rounding and scars due to perforating a semi-hard abrasive material on the proximal tip of tool n°B.

**Fig 13:**
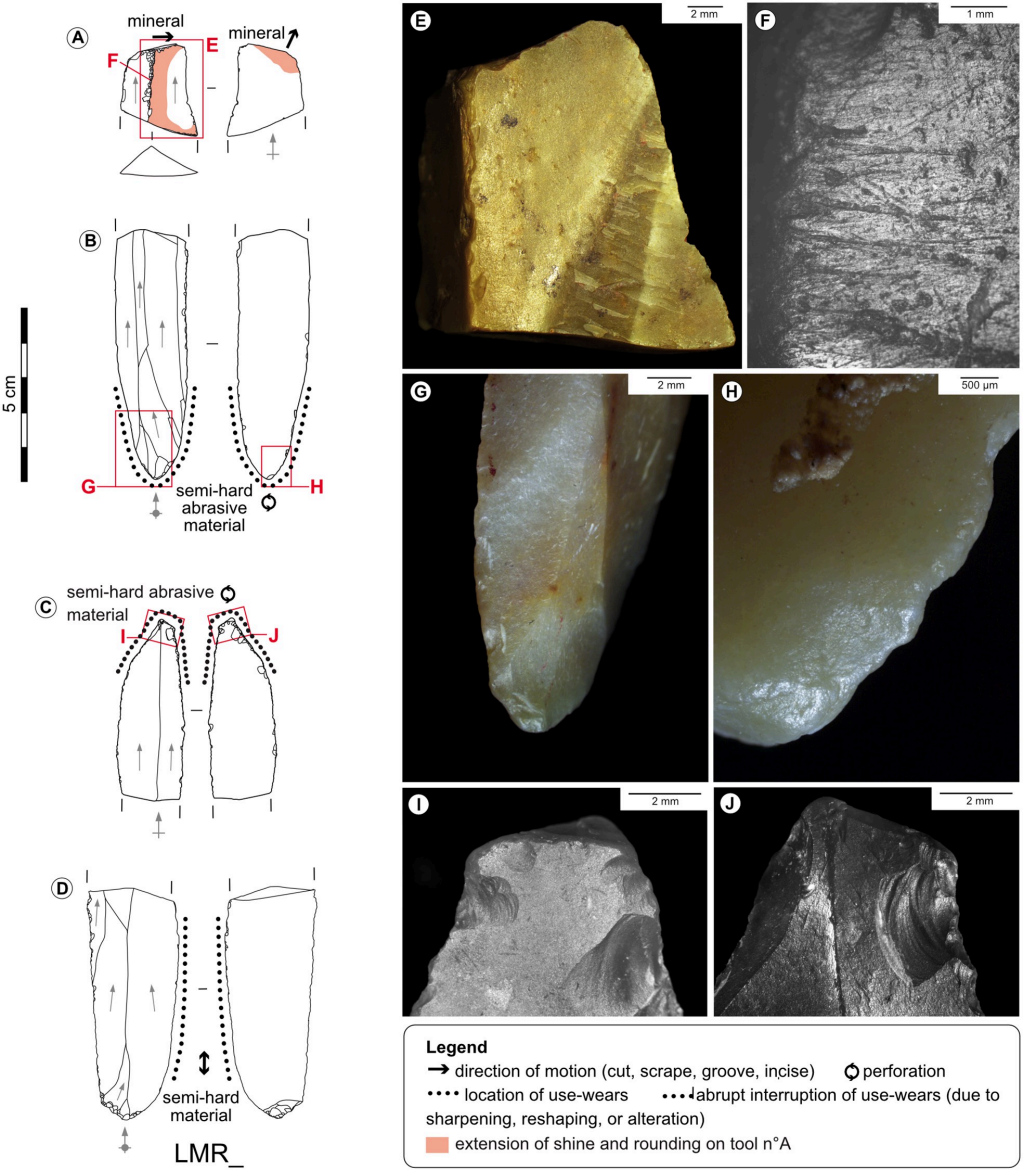
Diversity of zones used on unretouched blades. A-Mesial fragment of blade from the Blanchard cave, layer B4. B-Broken blade from the Blanchard cave, layer B3. C-Broken blade from the Blanchard cave, layer B4. D-Broken blade from La Marche. E, F-Traces of mineral scraping on the ridge of tool n°A, credits: Gauvrit Roux, 2019. G, H-Perforating traces on the proximal end of tool n°B. I, J-Perforating traces on the distal end of tool n°C.

In both sites, the stone tools were used to perform a wide diversity of activities related to acquiring and transforming animal, vegetal, and mineral resources (Figs 9–13, Table 1). The blades and the bladelets were used for distinct spheres of activity: the backed bladelets were employed in the hunting activity (see below) and the blades were involved in the domestic and artistic spheres [66, 67, 70, 73].

**Table 1:** Synthesis of the functional data for la Marche and the Blanchard cave in number of tools. S/SA: soft or soft abrasive worked material. SH/SHA: semi-hard or semi-hard abrasive worked materials. H/HA: hard or hard abrasive. *Including double tools and tools on truncated, retouched or thinned and truncated blade. All the levels of the Blanchard cave are presented together because of the high similarity of results between them.

Blank	Type	Blanchard cave										La Marche										
		Nb analysed	Projectile	Hide	Butchery	Osseous material	Vegetal	Mineral	S/SA	SH/SHA	H/HA	Nb analysed	Projectile	Hide	Butchery	Osseous material	Vegetal	Mineral	S/SA	SH/SHA	H/HA	Undet.
Burin spall		6		2			1					12		1			2	1	1	3		1
Flake	Burin											1									1	
	Burin-*pièce esquillée*	1									1											
	Endscraper	1		1						1												
	Bladelet core											1										
	*Pièce esquillée*	6									6	1									1	
	Unretouched	2								1												
Undet.	Backed piece											1										
	*Pièce esquillée*	3				1					3											
Blade	Beak*	3		1		1				1		29		7		2	3	1	3	9	7	1
	Beak-burin	2		1					1			3		2			1		1	1	2	
	Burin*	28		3				2	1	11	6	50		5		3	3		1	9	16	1
	Burin-*pièce esquillée*	3		1				1			3											
	Endscraper*	59		46			5	2	10	12	12	75		62	3		10		13	3	11	3
	Endscraper-beak	1								1		2		2				1			1	
	Endscraper-burin	30		23	2			1	6	6	6	12		10			1			3	3	
	Retouched and/or truncated	4		1				1			1	7						1	1	2	1	1
	*Pièce esquillée*	7									7											
	Unretouched	11			1		1	2	1	4	1	8		1			2			1		
Bladelet	Backed bladelet	173	104									181	126									
	Microperforator	2								2		12						3	1	5		1
	Microperforator on backed or truncated bladelet											1								2		
	Unretouched	8		1						3		6										
Bladelet core		1										2									1	
Total		351	104	80	3	2	7	9	19	42	46	404	126	90	3	5	22	7	21	38	44	8

The blades are highly valued items that have long use cycles and are, as often during the Upper Palaeolithic, a reserve of raw material and useful cutting edges [74, 75]. The strategies employed to prolong the life of tools or edges are varied:

The shaping of double and composite tools is relatively frequent as it concerns 9% of the tools on blade at the Blanchard cave, and 16% at La Marche. These tools can associate on one blank an endscraper, a burin, a beak, or a truncation (Figs 10 and 11). Double burins and endscraper-burins are the most frequent association; in this last case, the chronology of retouch removals and the interruption of use-wears on the endscrapers by negatives of burin spalls show that the burin is typically shaped after the shaping and use of the endscraper. This recurrent chronology suggests that the activity performed with endscrapers had a specific technical value. The frequent association of tools on the extremities of blades likely aimed at taking advantage of blades length and had an economical value.

Sharpening consists in rejuvenating the worn-out working edge by the same retouch technique used to shape it, in order to maintain its efficacy without changing tool. It is evidenced on endscrapers, burins, and lateral edges, where the most recent retouch removals (scars or spalls) occasionally cut through use-wears (Fig 11). The successive sharpenings of an edge necessarily provokes length reduction of the tool, and can cause modification of the edge angle (e.g., from semi-abrupt to abrupt), and of the edge delineation (e.g., from convex to rectilinear) [61, 67, 73, 74, 76–78].

Recycling can take many forms (i.e., reuse, reshaping) and refers to a change of the initially intended function of the tool; it can involve a modification of the tool’s shape [66, 74, 75]. Recycling concerns in particular endscrapers, whose distal end was first used to scrape hide, and then occasionally used to strike a hard material before discard (12% of the distal ends at La Marche and 17% at the Blanchard cave). The second use deeply modified the shape of the edge by causing crushing and large overlapped scars on both faces of the working edge. This chronology supports the abovementioned idea that the task performed with the extremity of endscrapers had a specific technical value, because the activities damaging the working edge (i.e., burin shaping, hard material percussion) always come last, before discard [66, 67, 73].

Reuse refers to the secondary use of an edge [75], and does not imply a modification of its shape. While the recycling corresponds to the rerouting of tools from their initial technical purpose (e.g., endscrapers are specialised in hide scraping, and rerouted to strike a hard material), the reuse can be part of the technical purpose for which the tool or the edge was intended (e.g., lateral edges of blades are multipurpose, and their reuse can be interpreted as part of their intended functional flexibility). Reuse is identified with certainty on the lateral edge of less than 5 blades per site, although it may concern a larger number of tools that cannot be recognised because of the complex overlapping of traces on the edges, often resulting in characterising mixed motions (i.e., cutting *and* scraping), and because of the post-depositional alteration of micro-wears, which restricts the interpretation to classes of hardness of worked materials.

Multiple uses refer to the utilisation of several zones of a tool; these zones include in both sites the extremity tools, lateral edges, fracture corners, unretouched distal ends, and more rarely butts or dorsal ridges. Multiple uses are common on blades (circa 43% of the used blades at La Marche and the Blanchard cave), revealing that the strategies employed to optimise the length of use of blades were fully integrated into mental and technical schemes [66, 67] (Figs 10 and 11, S3 and S4 Tables).

Magdalenian blades are frequently fractured [76, 79–82], and several elements suggest that at least part of them were broken intentionally, especially endscrapers: the high rate of broken endscrapers (90% at the Blanchard cave and 87% at La Marche), the preferential location of breakages on the proximal end (76% at the Blanchard cave and 72% at La Marche), and the presence of some rare impact points on the edge of the breakages. Intentional fracturing can be related to economic purposes (breakage allows to multiply the number of extremities on which tools could potentially be shaped), or to ergonomic requirements (breakage allows to reduce the slight curvature of the profile of blades and facilitates their insertion into a haft). Analysis of the chronology of retouch and use-wears in relation to fractures reveals that broken blades typically have the following history: first, the long lateral edges were used, or retouched and used, then the blank was fractured, later a tool was shaped on an extremity of the blank; this tool was then used, often sharpened in the case of burins and endscrapers, and endscrapers were occasionally recycled before being ultimately discarded.

These data show that blades were highly valued items with long biographies. The allochthonous flint procurement strategies may have encouraged both the segmentation of the process of production of blades, and their intensive use in order to reduce the economic cost of transporting blocks or blanks from the Grand Pressigny flint sources. This can participate in explaining why blades had a specific socio-economic status among the toolkit. For instance, blades management strategies are not recognised on microliths whose management differs, possibly due to the reduced cost of transporting smaller and lighter tools and cores, and to the ergonomic constraints of hunting weapons.

### 5.3. Projectile designs

**Fig 14:**
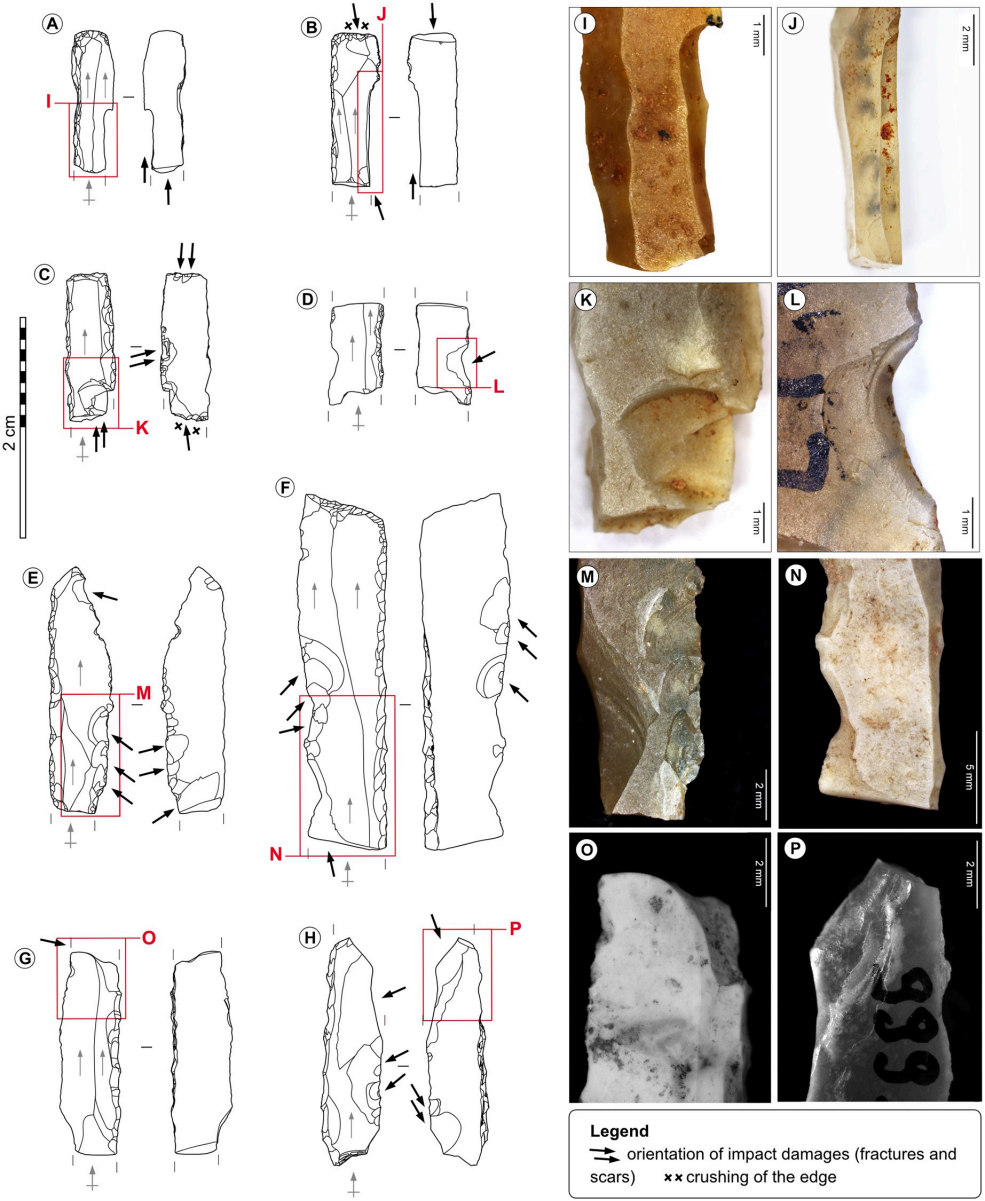
Impacted backed bladelets. A-Truncated simple backed bladelet from the Blanchard cave, layer B2. B-Double backed bladelet from the Blanchard cave, layer B4. C-Truncated double backed bladelet from the Blanchard cave, layer B3. D-Simple backed bladelet from the Blanchard cave, layer B5. E, G-Simple backed bladelet from La Marche. F, H-Truncated simple backed bladelet from La Marche. I-Burin-like spin-off fracture on tool n°A. J-Burin-like spin-off fracture on tool n°B. K-Facial spin-off fracture on tool n°C. L-Large isolated impact scar on tool n°D, credits: Gauvrit Roux, 2019. M-Large overlapped impact scars on tool n°E. N-Twisted bending fracture on tool n°F. O-Burin-like fracture on tool n°G. P-Oblique bending fracture interrupted by a snap fracture on tool n° H.

The backed bladelets, which are occasionally truncated or appointed, show exclusively impact damages due to their use as projectile inserts (lateral impact scars, microscopic linear impact traces, and burin-like, bending, spin-off impact fractures) [70, 83–85] (Fig 14, S5–S8 Tables). The near systematic fracturing of these microliths after the abrupt and rectilinear retouch of the back, the snap morphology of most fractures, and the scarcity of proximal fragments suggest their intentional breakage to obtain thin tools that have a straight and regular profile with parallel lateral edges.

Several elements suggest that the backed bladelets were hafted to projectile points, and that at least part of them were positioned laterally (i.e., microliths distant from the impact point) with their back fixed to the projectile. At both sites the lateral impact scars are abundant, a feature which is experimentally specific to lateral inserts, since the slashing/tearing edge of lateral lithic inserts seemingly absorbs most of the constraints of the impact [70, 84]. The orientation of the impact damages suggests that the impact forces stroke the inserts parallelly to their length: the impact striations are parallel to the back, the impact fractures are oblique or parallel to it, and the lateral impact scars are oblique to it. The occasional presence of residues on the back and on the surfaces near the back of part of the microliths suggest that the back was fixed to the projectile point [66, 70]. In addition, both Magdalenian examples of composite points from the Blanchard cave and Pincevent [86, 87] show that the microliths or fragments of microliths were positioned laterally in the grooves of osseous points.

Despite an apparent similarity of the functioning of microliths, the patterns of impact fractures differ deeply between sites (S5–S8 Tables): at La Marche, the bending and burin-like fractures are the most common impact breakages (94% of the tools with impact fractures), whereas, at the Blanchard cave, the spin-off fractures dominate (56%). These differences could be related to distinct hafting modalities and match the inter-site variations of the main morphologies of osseous projectile points [2, 9, 10]: the short single-bevelled Lussac-Angles points, and the long double-bevelled points from La Garenne allow the hafting of a different number of lateral inserts [70].

### 5.4. Subsistence strategies based on the exploitation of animal resources

The vestiges of the hunting activity, which include the faunal remains and “the instruments of predation[,] are of a disconcerting profusion during [all] the Upper Paleolithic” [88]. The hunting activity is therefore particularly visible compared to other crafts for which few data are available, such as tools used to manufacture ornaments or to work vegetal materials.

The exploitation of animal resources appears to have had a dominating role in the economy of both the analysed sites: first, the faunal remains, the osseous tools and the discards of the osseous production (bone, teeth, antler, shells) are abundant, and, second, the flint tools were mostly used to acquire and process the faunal resources (Table 1): the backed bladelets are the most numerous tools and were most likely used to acquire animal resources. Numerous tools on blades were used to process these resources for alimentary or technical purposes. The best-represented activities identified on the blades are hence related to hide working (respectively 35% and 33% of the pieces with use-wears from La Marche and the Blanchard cave). The second best represented worked materials are the undetermined hard materials (respectively 13% and 15% at La Marche and the Blanchard cave), which likely include osseous materials. Osseous material working is under-represented on the flint tools from both sites (about 1% in the two caves), whereas the bone industry is abundant. In addition, at La Garenne, at least part of the manufacturing wears on the antler artefacts are due to burin bevels [89]. The osseous material working is probably undetectable because of micro-wear alteration, as in other Magdalenian contexts [80, 90]. Certain operations such as butchery are also highly under-represented on flint tools (about 1% in the two sites); these traces are only identified on unretouched lateral edges. Several factors can contribute to explaining the rarity of butchery wears: sampling strategies (few unretouched artefacts analysed), intensive use of blades (use-wears may have been removed when lateral edges were retouched), short-duration uses of the edges (they generate tenuous traces that do not allow to determine the worked material) [65, 66, 91–93], taphonomy bias (mechanical and chemical alteration), or segmentation of the butchering process (realisation of some operations outside the caves). Notwithstanding the scarcity of butchery traces on stone tools, the richness of the faunal assemblages and the analysis of the traces on bones show that animal processing had an important role at the sites. Several butchering operations are indeed evidenced on the reindeer, horse, bovines, ibex, saiga antelope, chamois, wolf, fox, and hare remains from the Blanchard cave. These operations include skinning, defleshing, disarticulating, bone scraping, and bone fracturing, operations which aimed at extracting a wide range of materials used for alimentary, technical, or symbolic purposes: the meat, the fat, the marrow, the tongue, the hide, the tendons, the horns, the horsehair, the antlers. Part of the butchering activity likely took place on the kill site, since the skeletal-parts representation indicates that certain species (reindeer, horse, bovines, ibex) were brought to the site in fleshy quarters [25].

At La Marche and La Garenne, the flint tools used to saw, scrape, or perforate wood represent respectively 6% and 2% of the artefacts with use-wears (tools on blade, unretouched blades, and burin spalls; Table 1), whereas the tools used to acquire or process animal resources total 61% of the assemblages, despite the abovementioned under-representation of butchery and osseous materials traces on stone tools. More generally, the available extensive traceological analyses of West European Upper Palaeolithic assemblages show that the tools related to vegetal-working are particularly rare [66, 74, 80, 94–98], and for all the period, the wood or fibre artefacts are scarce [99–102]. Specific Upper Palaeolithic stone tools could have been dedicated to vegetal working, such as the Noailles burins [103, 104]; functional interpretations regarding these tools however vary considerably from site to site [105–109] probably due to the poor development of traces, and their technical purpose thus remains highly problematic. The gap of data regarding vegetal working may correspond to conservation issues with, besides the degradation of vegetal remains, the differential alteration of micro-wears of vegetal on stone tools [80]. In addition, the macro-wears of wood rarely allow to identify this worked material because the rounding and the striations it causes are often tenuous. On the reverse, contact materials such as dry hide or mineral may, to a certain extent, be identified through macroscopic attributes even when the polish is altered. Further methodological developments are necessary to better understand the taphonomic processes at stake in the differential alteration of vegetal polishes on stone tools. The taphonomic aspects may not entirely explain the near-absence of use-wears of vegetal for tens of thousands of years in highly diverse sedimentary contexts [110, 111]. The scarcity of direct and indirect evidences of plants processing may also indicate that flint tools were rarely involved in working these materials; the use of manual strength or of tools made of perishable matter can be hypothesised [66]. The Upper Palaeolithic yields an abundant bone and antler industry and one may wonder to which extent the richness of these osseous assemblages can be related to the rarity of wood tools and woodworking traces [110]. Conversely, woodworking with flint tools rises after the Magdalenian period, during the Holocene climate warming [92, 95] and matches an impoverishment of the osseous industry.

Pending the development of functional analyses on large and well-preserved Upper Palaeolithic assemblages, the place of the vegetal in the economy remains widely inaccessible. The under-representation of remains and use-wears of vegetal cannot, however, be considered as an indicator of the role of this material in the Palaeolithic economy as it is essential in composite tools manufacturing: projectile shafts, scrapers or knife hafts potentially made of wood, and certain fibres may have been used to ligature lithic inserts. To the richness of osseous points, lithic projectile inserts, scrapers, and cutting blades must have corresponded a high quantity of vegetal shafts and hafts, or even foreshafts and links, of which no evidence is preserved.

Because of the perishable nature of certain archaeological materials, it is not possible to appreciate the relative importance of vegetal and animal resources in the subsistence strategies. It is clear that the exploitation of animal resources has a considerable role in the Magdalenian economy and that numerous ramified *chaînes opératoires* stem from it, such as projectile manufacturing, hunting, butchery, hide, antler, bone, or tendon working. Defining the technical parameters that take part in fauna exploitation thus takes on a privileged interest to better understand the Palaeolithic techno-economical and societal dynamics.

### 5.5. Approaching the function of the sites

Defining the functioning of archaeological tools provides access to the activities represented at a site, and to certain steps of technical processes. By replacing these elements into their system and linking them to the other categories of productions, it is possible to approach the economic function of a site on the condition of considering coherent levels of human occupation. The archaeological contexts considered here are palimpsests composed of successive undifferentiated occupations, as often in Palaeolithic cave occupations. The palethnographic interpretations that can be reached are therefore limited.

Several elements however suggest that both caves were mostly occupied for relatively long periods of time, although it is impossible to establish the precise timing of occupation. This can reflect several site functions which cannot be precisely determined due to taphonomic issues and early excavations: base camp, residential site, and aggregation site [112–116].

The archaeological assemblages are particularly substantial, with tens of thousands of knapped flint artefacts, colouring fragments and faunal remains. The portable art and the ornament elements are amongst the richest of the regional EMM and of the EMM in general [3, 8, 15, 16, 36–38, 55]. The antler or bone finished products are abundant in both sites and are, like the portable art and the ornaments, among the richest of the period [3, 9, 10]. The sole quantity of archaeological material does not allow to determine site function, in particular in the limited space of caves where “the repetition [of the] installations in a context of slow sedimentary covering can produce a misleading effect of abundance of remains” [115]. However, the assemblages considered are not only rich, they are also diversified and the osseous and lithic productions show the realisation of numerous steps of the *chaînes opératoires*: manufacturing, use, resharpening, repairing, recycling, until the discard of tools (see above, Fig 2). For instance, the osseous industry from the Blanchard cave shows several steps of manufacturing of projectile points, from the extraction of the rods (waste products, blank drafts), to the repairing of some objects [117–121]. The *chaînes opératoires* are segmented at different levels according to the economic activity or category of product that is considered (e.g., blades mainly produced outside the caves *vs* bladelets produced at the sites). Nonetheless, the acquisition step of the raw materials (flint, shells, fauna, colourant, and, at La Marche, limestone slabs) is systematically absent [8, 25, 37, 60, 122, 123]. In this way, the hunting weaponry damaged during the acquisition of faunal resources was likely brought back to the sites to be repaired in anticipation of new hunting expeditions.

The stone tools are typologically varied and were used for a wide range of activities related to the hunting, domestic, and artistic spheres. The blades were intended for domestic and artistic activities and the bladelets were dedicated to the hunt. The flint tools were mostly used to acquire or transform the animal resources in both sites. The endscrapers used for hide working are abundant and were used for several steps of hide processing [66, 73]. They correspond to standardised processes that can be relatively long to set up (e.g., drying thick hides), reinforcing the idea of prolonged occupations of the sites. In parallel, few flint tools show traces of mineral engraving. The two caves appear to have been living spaces where numerous activities took place besides the production of portable and cave art: production of bladelets, manufacturing and repairing of hunting weapons with replacements of the broken inserts, butchering, processing of hides that were dry, moistened, with additive, or with abrasive, manufacturing of ornaments made of shells, and of elements originating from the hunt such as teeth and hyoid bones…

Seasonality data support the hypothesis of prolonged occupations of the sites and of the region: the killing seasons of reindeers and horses cover several months in both sites (Table 2) [25, 54]. A prolonged occupation of the neighbouring Roc-aux-Sorciers has previously been proposed for the EMM layers, based on the quantity and the diversity of artefacts, and the seasonality data [15, 28]. This is supported by the wide range of activities performed at the site [74, 124].

**Table 2:** Seasonality data for the Early Middle Magdalenian sites of west-central France. Black dots: certain; Empty dots: uncertain. Data after Bayle et al. (2009), P. and J. Bouchud in Pradel (1958), Valensi (2010), Valensi and Boulbes (2018).

Site	Cave	Level	Species	Jan.	Feb.	March	April	May	June	July	Aug.	Sept.	Oct.	Nov.	Dec.
La Garenne	Grand Abri	Ens. B	Reindeer	**●**	**●**	**●**							**●**	**●**	**●**
			Horse	**●**										**●**	**●**
	Blanchard cave	B3	Reindeer	○	○	○	○	●	●	○	○	○	●	**●**	**●**
			Horse	○	○									○	○
		B4-C1	Reindeer	**●**	**●**	**●**	**●**	○	○					○	●
			Horse	○	○									○	○
		B5-C2	Reindeer	○	○	○	○								
La Marche		3	Reindeer	●	●	●	●		●	●	●	●	●		●
Roc-aux-Sorciers	Bourdois rock shelter	D	Reindeer		●	●							●	●	
			Horse	○	●			●	○	○	●	●		●	●
		E	Horse		○	○	○	○	○	●	●				
	Taillebourg cave	C	Reindeer					●	●	●	●	○	●	○	
			Horse			○	○	●	○	○			●	●	●
		D	Reindeer	●	●	●	○	●	●	●	●	○			
			Horse	●	○	○		●	●	●	○		●	○	○
			Saiga	●	●								●	○	
			Bison					●						●	●

At the Blanchard cave, the killing season is often restricted to the bad season and the beginning of the good season (layers B4-C1 and B5-C2), as generally observed at the Grand Abri (assemblage B). This type of occupation during the bad season is reminiscent of the meridional sites of the Middle Magdalenian of the Montagne Noire, assimilated to winter residential sites: the Gazel cave in Sallèles-Cabardès, Canecaude in Villardonnel, Bize in Bize-Minervois, the Crouzade in Gruissan [125, 126]. The occupation of the Roc-aux-Sorciers was occasionally limited to the good season (the layer E of the Taillebourg cave and the layer C of the Bourdois rock shelter), indicating a seasonality of occupation almost reversed from what is observed in most layers of the Blanchard cave, and therefore potentially different mobility systems or site functions.

The killing seasons represent almost all the periods of the year at the Blanchard cave (layer B3), at La Marche, and the Roc-aux-Sorciers (the layer D of the Taillebourg cave and the layer D of the Bourdois rock shelter) [28, 127]. This could suggest that the occupation of these sites was not only seasonal; however, the killing seasons probably cover several occupation episodes because of the palimpsest effect.

Considered together, the seasonality data available for three EMM sites of west-central France show that human groups (and probably certain ungulates herds) were present all year long in the Vienne, the Creuse, and the Gartempe Valleys. This evidences a strong regional implantation of human societies and participates in explaining the specificity of the EMM in west-central France.

## 6. Regional socio-economic interactions

Apart from the recent discovery of the open-air site of the Route de la Roche in Solutré [128], the totality of the EMM sites is in caves or rock shelters. In the cavities of west-central France, as well as in numerous contexts of southwestern of France, the blade-knapping generally occurred outside the sites, the bladelets were produced at the sites, and both blank types were mostly made of good quality allochthonous flints. This is notably the case at La Marche (Primault, com. pers.), the Blanchard cave [60], and the Taillis-des-Coteaux [129], where most exploited flints come from the Upper Turonian of the Grand-Pressigny region, and the Lower Turonian of the Indre and Cher Valleys. This suggests an interdependency between the cave and rock shelter sites (whose occupation was seemingly prolonged, where the acquisition step of flint is absent, and where by-products of the blade production are rare) with the numerous open-air sites located on the Grand Pressigny flint deposits, which are suspected to have been sites dedicated to flint-knapping [130–132]. These evidences of Magdalenian presence cannot, however, be precisely dated, because the sites are rarely stratified, the retouched tools are rare, there is no bone industry, and no faunal remains. The development of thematic surveys paired with the multidisciplinary study of the assemblages will therefore be necessary to better understand the functioning of the sites in a regional network and their economic complementarity.

The strong similarity of circulation networks of allochthonous raw materials (flint and shells) of the MLA and MN sites [5, 8, 60, 122, 133] indicates the sharing of an economic territory where diverse forms of social and economic interactions may have taken place, through the circulation of goods, people, or ideas. The good quality flints from the south of the Paris Basin (i.e., Grand Pressigny flints) were systematically and abundantly exploited during the EMM in west-central France. These raw materials also circulated on long distances towards the south [1, 134] and the Massif Central [7]. This large-scale diffusion necessarily led to new inter-regional socio-economical interactions.

These interactions may have 1/participated in reinforcing the unity of the EMM as part of an economic cooperation that generated social links over a wide area [135], or 2/favoured the differentiation of social units who affirmed their identity facing neighbouring groups, notably in relation with a competition for the use of a same economic territory [136].

The abundance, the diversity, and the specificity of the markers of the MLA and of the MN in west-central France indeed suggest that the region was a crossroads where certain differences between technical traditions were exacerbated. This exacerbation of differences by means of “productions with an ostentatious character” [137], which include art, ornaments, and probably projectile design, could then be related to mechanisms of territorial affirmation [15].

This suggests that the MLA and the MN are cultural units whose territory is identifiable. However, the methodological tools available do not allow to affirm it because the EMM traditions are still widely defined from specific objects acting as index fossils. This approach is a common and useful basis for reflection on the Prehistoric chronological frame, but it can be problematic for several aspects: first, the markers that are used do not reflect technical systems as a whole since they are chosen among diversified material productions that are partly similar between sites. Then, part of the markers is found isolated in the sites outside west-central France and their place in the techno-economic, social, and symbolic systems cannot be the same as when they dominate the osseous, lithic, art, or ornamental assemblages (Fig 1). Besides, certain assemblages of the Gironde (rock shelter 1 at Moulin-Neuf) and of the Massif Central (e.g., Le Rocher de la Caille, Enval, Le Rond du Barry) do not yield any classic markers, which underlines the necessity of using other methodological tools to grasp the diversity of the regional expressions of the EMM [134, 137]. Last, all the markers do not have the same geographical distribution (Fig 1) [3, 46]: part of the elements characterising the MLA and the MN are specific to west-central France, such as the horse teeth engraved with geometrical patterns, which are associated with the MLA [41, 133]. Other productions are found far beyond western France but are concentrated in the sites of west-central France, such as the Lussac-Angles points or the Navettes (Fig 1). What criteria should then be used to identify a tradition and its cultural territory? Choosing some criteria and extracting them from the technic and symbolic system to which they belong would be meaningless. In addition, the geographical distribution of one or several objects does not necessarily reflect a cultural territory [138, 139], since “the types of [objects] have no demonstrable relation with social units aware of their identity” [140]. However, it is precisely the awareness and the affirmation of belonging to a cultural unit that allows to define this unit [138], and it is not possible to reach this emic conception because of the very nature of the archaeological material.

The cultural dimension of the MLA and the MN remains therefore out of reach, and their territory cannot be delimitated. It is however possible to enhance the reflection on the socio-economic processes at stake if these units are considered as traditions defined as “system[s] of choices and prescriptions in the technic and symbolic activities which are accessible in archaeology” [137], where techniques thus carry information on social interactions.

The differences between the EMM technical traditions are often stressed. Nevertheless, in every technic and artistic field, there are strong similarities which are the expression of a community of practice, and attest of marked interactions between the MLA and the MN. The unity of part of the material production over a large area furthermore links these regional dynamics to the whole of the wide territory of extension of the EMM.

The ornamental productions [8, 133], the osseous industry [2, 141], and the artworks [15, 16, 39, 142] attest both of technic or symbolic systems partially differing between the MLA and the MN, and of a regional or even inter-regional unity. For instance, the representation of human faces is a recurring theme in the EMM of west-central France, whereas it is almost absent outside the region, and is particularly rare in all the European Palaeolithic art. This theme unites the traditions of west-central France and contributes to the regional specificity. In parallel, the style (i.e., realistic *or* schematic), and the blanks (i.e., rock *or* osseous material) of the human representations participate in distinguishing the MLA from the MN [142, 143]. Besides, certain art productions show the existence of permeability and mutual influences between traditions. This is the case of horse representations at the Taillis-des-Coteaux, whose style is intermediary between the tradition with navettes and with Lussac-Angles points [39].

The lithic sub-system contributes to evidencing the strong interactions that existed between the EMM traditions. The methods and techniques used to produce the standardised blades and bladelets are common to west-central and southwestern France, and to certain assemblages of the Massif Central. They are marked by a highly normative blade production and the polymorphism of the production processes of bladelets [7]. The blades were detached from large flaking surfaces, the rhythm was unipolar and the progression semi-rotating, with a transversal management of angles and convexities, and the bladelets were produced on prismatic or pyramidal cores with a unipolar or unipolar preferential rhythm, the progression on the large flaking surface was rotating or semi-rotating on blocs, or on the slice of thick flakes, plus possibly on the upper face of blades at La Marche [1, 3, 6, 7, 19, 46, 124, 129, 137, 144].

The comparison of the functioning of the stone tools from La Marche and the Blanchard cave also shows significant similarities in tools management, worked materials, and technical gestures. This is the expression of strong technic norms, part of which are common to many Upper Paleolithic sites of western Europe, such as the intensive use of blades which are the object of several cycles of use and sharpening.

The typology of the microliths could however mark inter-regional differences: the scalene bladelets are specific to southwestern France and northern Spain, and truncated backed bladelets are systematically found in the assemblages of west-central France, both in MLA and MN contexts [1, 3, 6, 46, 144]. Certain backed bladelets with an oblique truncation of west-central France could be assimilated to the scalene morphotype because of the open angulation formed by the intersection of the truncation and the back, although the preferential lateralisation of the back is absent. This is the case at La Marche, at the Roc-aux-Sorciers [124], at the Fadets, or at Montgaudier, Paignon rock shelter [19], and it may be related to borrowing or imitation phenomenon. These typological differences cannot be fully understood without a functional approach considering that similar shapes of tools may be associated with different functions, and reversely [145]. The scalene bladelets have not yet been examined with the traceology methods and their functioning thus remains unknown. The development of such studies is crucial to understand the technical purposes underlying the diversification of microlithisms during the Magdalenian.

Besides inter-regional typological variations of microliths, techno-functional data allow to evidence functioning differences between two sites of west-central France yielding truncated backed bladelets; these differences are probably correlated to distinct projectile designs, and to variations of the morphology of the osseous points. Further studies are necessary to examine if the correlation between the morphology of the typical MLA and MN osseous points and the patterns of impact fractures of backed bladelets is found in other EMM contexts. Preliminary comparisons suggest that the use of distinct projectile designs in west-central France is not correlated to differences of environment or hunted ungulates [25, 54], but could be part of the assemblage of ostentatious productions (art, ornaments) distinguishing the two technical traditions.

## 7. Conclusions

During the EMM, west-central France is a crossroads of at least two technical traditions which mutually influenced one another in many fields: art, ornaments, osseous industry, probably subsistence strategies, and the procurement, production, use, and management of stone tools. We argue that the marked regional implantation of human groups in the Vienne, the Creuse, and the Gartempe Valleys is likely a major factor to understand the specificity of the EMM expressions in the area, as well as the sharing, in the same economic territory, of technical norms and of part of the system of symbolic representation. This strong territoriality (effective and symbolic) is evidenced through the high density of cave and rock shelter sites (although this is probably partly an effect of the state of the archaeological research), which were likely long-term occupations (at least several months a year with recurrent occupations), and where a wide range of activities took place, related to the domestic, artistic, and hunting spheres. This implies that the ungulate herds were present for long periods every year, or that there were strategies to conserve meats, such as drying or freezing, of which we have no evidence. All the codes are not shared in the regional space, and traditions are distinguished through ostentatious productions including art, ornaments, and the design of hunting weapons, which probably “crystallise mechanisms of otherness and identity” [143].

The circulation networks of the raw materials from the south of the Paris Basin to the Aquitaine Basin and the Massif Central, the sharing of the blade and bladelet *débitage* concepts, and the possible circulation of certain isolated objects to the south such as Lussac-Angles points, indicate the interconnection of west-central France with other regions. The circulation networks of flints and shells extend far to the east until Poland [146], showing the wide extension of the communication web across Europe.

Many aspects should be further explored to understand more precisely the regional and inter-regional dynamics, regarding in particular technical transfers (sharing or not of designs and techniques of use of stone tools), subsistence strategies (techniques and tools used in acquiring and processing animal and vegetal resources in relation with environments), and the functioning of sites in their regional network. The development of network analysis [3, 147, 148] and niche modelling [149] paired with extensive techno-functional analysis of reliable and well-preserved assemblages will be crucial to gain a fuller comprehension of the socio-economic organisation and spatial structuration of Magdalenian hunter-gatherer nomadic societies.

## Supporting information

S1 Fig Distribution of the width and thickness of retouched blades, retouched bladelets and unretouched blanks at La Marche and the Blanchard cave. (TIF)

S1 Table Typological composition of the corpus included in the technological analysis of the lithic assemblage of La Marche. (PDF)

S2 Table Typological composition of the corpus included in the technological analysis of the lithic assemblage of the Blanchard cave. (PDF)

S3 Table Number of used areas (UA) per tool at La Marche. Without backed bladelets. Modified after Gauvrit Roux (2019).
(PDF)

S4 Table Number of used areas (UA) per tool at the Blanchard cave. Without backed bladelets. Modified after Gauvrit Roux (2019).
(PDF)

S5 Table Condition of the proximal edge of backed bladelets at the Blanchard cave. Modified after Gauvrit Roux (2019).
(PDF)

S6 Table Condition of the distal edge of backed bladelets at the Blanchard cave. Modified after Gauvrit Roux (2019).
(PDF)

S7 Table Condition of the proximal edge of backed bladelets at La Marche. Modified after Gauvrit Roux (2019).
(PDF)

S8 Table Condition of the distal edge of backed bladelets at La Marche. Modified after Gauvrit Roux (2019).
(PDF)
