# Supplementary figures and images for: Socio-economic dynamics of Magdalenian hunter-gatherers: Functional perspective
Source: PLoS One. 2022 Oct 5;17(10):e0274819. doi: 10.1371/journal.pone.0274819 (PMC9534454; doi:10.1371/journal.pone.0274819)

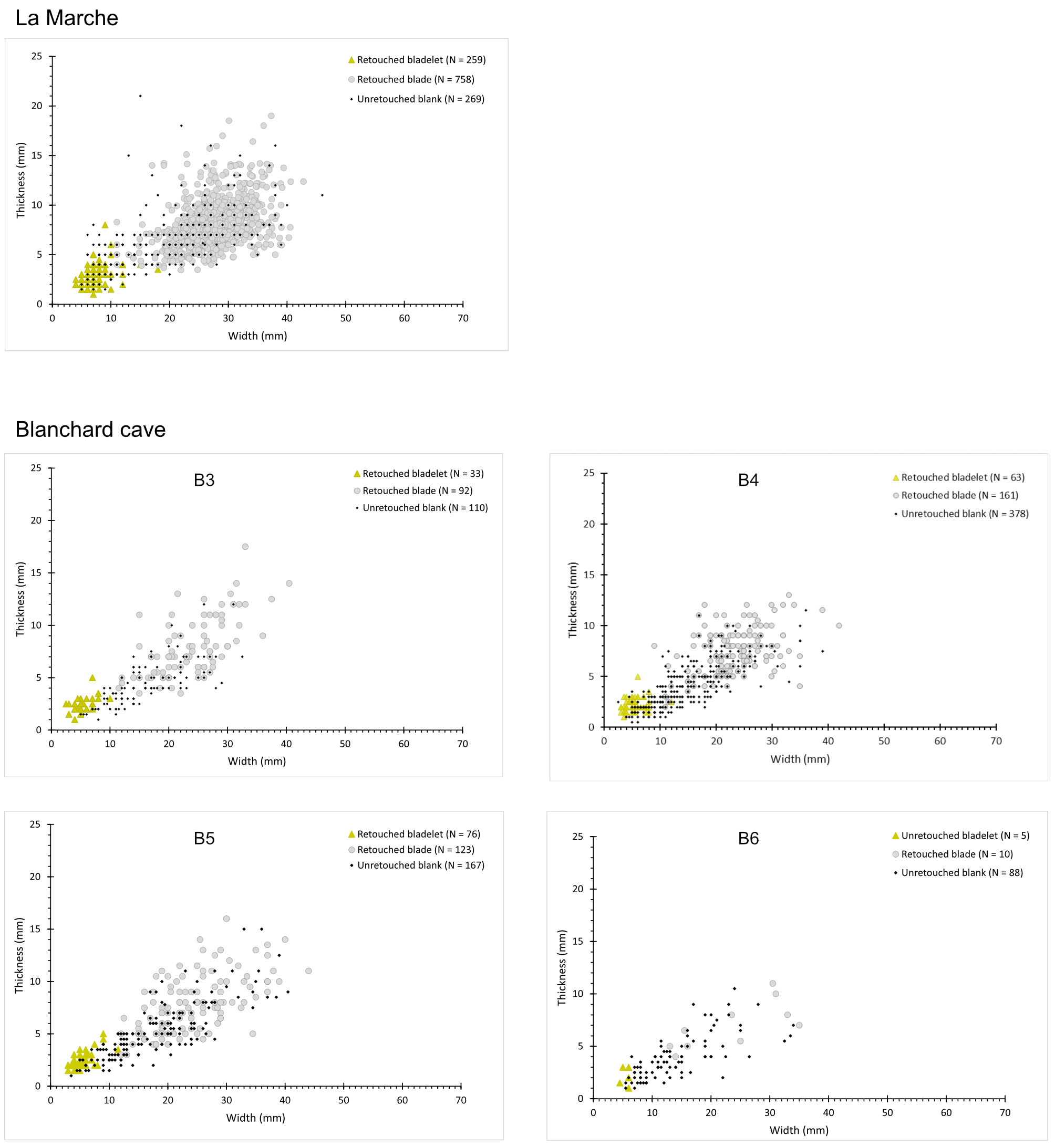

Supplement: S1 Fig — (TIF) [file pone.0274819.s001.tif]
