# Supplementary material for: Socio-economic dynamics of Magdalenian hunter-gatherers: Functional perspective
Source: PLoS One. 2022 Oct 5;17(10):e0274819. doi: 10.1371/journal.pone.0274819 (PMC9534454; doi:10.1371/journal.pone.0274819)
Supplement: S1 Table — (PDF) [file pone.0274819.s002.pdf]

Blank	Type	Nb	%
Burin spall	Backed burin spall	24	0,43
	Microperforator	2	0,04
	Unretouched	315	5,66
Flake	Beak	2	0,04
	Burin	5	0,09
	Chip < 1 cm	11	0,20
	Endscraper	3	0,05
	Retouched flake	1	0,02
	Unretouched	249	4,47
Undetermined	Backed piece	1	0,02
	Burin	1	0,02
	Endscraper	1	0,02
	Pièce esquillée	1	0,02
	Unretouched	12	0,22
Blade	Beak	367	6,59
	Double beak	12	0,22
	Beak on truncated blade, or on truncated and thinned blade	8	0,14
	Beak-burin	16	0,29
	Burin	452	8,12
	Double burin	72	1,29
	Burin on truncated blade, or on truncated and thinned blade	10	0,18
	Endscraper-burin	121	2,17
	Endscraper-beak	41	0,74
	Endscraper	583	10,47
	Double endscraper	29	0,52
	Endscraper on truncated blade, or on truncated and thinned blade, or on notched blade	16	0,29
	Retouched, truncated, thinned and truncated, or notched blade	215	3,86
	Pièce esquillée	4	0,07
	Unretouched	354	6,36
Bladelet	Backed bladelet, truncated backed bladelet, denticulated backed bladelet, appointed backed bladelet	2050	36,82
	Microperforator	30	0,54
	Microperforator on truncated bladelet	1	0,02
	Microperforator on backed bladelet	2	0,04
	Notched bladelet	2	0,04
	Unretouched	449	8,06
Core	Core	105	1,89
	Beak on core	1	0,02
Total		5568	100
