# Supplementary material for: Socio-economic dynamics of Magdalenian hunter-gatherers: Functional perspective
Source: PLoS One. 2022 Oct 5;17(10):e0274819. doi: 10.1371/journal.pone.0274819 (PMC9534454; doi:10.1371/journal.pone.0274819)
Supplement: S2 Table — (PDF) [file pone.0274819.s003.pdf]

Blank	Type	Level				Total	%
		B3	B4	B5	B6		
Burin spall	Backed burin spall	2				2	0,14
	Unretouched	3	7	1		11	0,78
Flake	Beak on truncated flake			1		1	0,07
	Burin	1	2	6		9	0,64
	Double burin			1		1	0,07
	Endscraper-burin	1		1		2	0,14
	Endscraper	1	1	1		2	0,14
	Pièce esquillée	7	2	1		9	0,64
	Unretouched		3	2	1	5	0,36
Undetermined	Burin			1		1	0,07
	Double burin			1		1	0,07
	Endscraper			1		1	0,07
	Pièce esquillée	1	5	2		8	0,57
Blade	Beak	2	2	2		6	0,43
	Beak-burin	1		1		2	0,14
	Burin	23	35	35	1	94	6,69
	Double burin	4	11	8	1	24	1,71
	Burin on truncated blade		3	3		6	0,43
	Burin-*pièce esquillée*	1	1			2	0,14
	Endscraper-burin	14	25	16	2	57	4,05
	Endscraper	34	64	40	1	137	9,74
	Double endscraper	3	5	5		13	0,92
	Endscraper on truncated blade		1	1		2	0,14
	Endscraper-beak		3	2		5	0,36
	Retouched or notched blade	3	19	17	2	2	0,14
	Truncated blade	8	9	5	3	25	1,78
	Pièce esquillée	3	5	1		9	0,64
	Pièce esquillée on retouched blade	1	1			2	0,14
	Undetermined		1			1	0,07
	Unretouched	72	213	112	44	441	31,37
Bladelet	Backed bladelet, truncated backed bladelet, denticulated backed bladelet, appointed backed bladelet	31	63	74	5	169	12,02
	Notched bladelet		1	1		2	0,14
	Truncated bladelet	1				1	0,07
	Microperforator		1	1		2	0,14
	Unretouched	41	158	58	45	302	21,48
Core			1			1	0,07
Total		258	642	401	105	1406	100
