# Supplementary material for: Socio-economic dynamics of Magdalenian hunter-gatherers: Functional perspective
Source: PLoS One. 2022 Oct 5;17(10):e0274819. doi: 10.1371/journal.pone.0274819 (PMC9534454; doi:10.1371/journal.pone.0274819)
Supplement: S3 Table — Without backed bladelets. Modified after Gauvrit Roux (2019). (PDF) [file pone.0274819.s004.pdf]

**Blank**	**Type**	**Nb of UA**								**Total**
		**0**	**1?**	**1**	**2?**	**2**	**3**	**4**	**5**	
Burin spall	Backed burin spall		1	3						4
	Microperforator	1				1				2
	Unretouched	3		3						6
Flake	Burin			1						1
	Core	1								1
	Pièce esquillée					1				1
Undetermined	Backed piece	1								1
Blade	Beak	6	1	15		2	1	1		26
	Beak on truncated blade						1		1	2
	Beak-burin					1	1	1		3
	Double beak					1				1
	Burin	11	1	16	1	4	1		1	35
	Burin on truncated thinned blade	3				1				4
	Burin on truncated blade	1		1					1	3
	Double burin	4		2		2				8
	Endscraper			39	1	21	5			66
	Endscraper on truncated blade					2	2	1		5
	Endscraper-beak					1	1			2
	Endscraper-burin	1		4	1	4	1	1		12
	Double endscraper					2	1	1		4
	Notched and truncated blade		1							1
	Blade with double truncation			1						1
	Retouched blade	1		1						2
	Truncated blade	1		1		1				3
	Unretouched	5		2		1				8
Bladelet	Microperforator	1	1	10						12
	Microperforator on backed bladelet			1						1
	Unretouched	6								6
Core on block		1				1				2
Total		47	5	100	3	46	14	5	3	223
