# Supplementary material for: Socio-economic dynamics of Magdalenian hunter-gatherers: Functional perspective
Source: PLoS One. 2022 Oct 5;17(10):e0274819. doi: 10.1371/journal.pone.0274819 (PMC9534454; doi:10.1371/journal.pone.0274819)
Supplement: S4 Table — Without backed bladelets. Modified after Gauvrit Roux (2019). (PDF) [file pone.0274819.s005.pdf]

Blank	Type	Nb of UA					Total
		0	1	2	3	4	
Burin spall	Backed burin spall	1	3				4
	Unretouched	2					2
Flake	Burin-*pièce esquillée*			1			1
	Endscraper			1			1
	Pièce esquillée			4		2	6
	Unretouched	1	1				2
Undetermined	*Pièce esquillée*			3			3
Blade	Beak		1	2			3
	Beak-burin		2				2
	Burin	5	12	3	1		21
	Burin on truncated blade		1				1
	Burin-*pièce esquillée*			2	1		3
	Double burin	2	3	1			6
	Endscraper		15	4	2		21
	Endscraper on truncated blade		1				1
	Endscraper-beak		1				1
	Endscraper-burin		13	14	2	1	30
	Double endscraper		16	16	3	2	37
	Retouched blade	1	2	1			4
	Pièce esquillée			7			7
	Unretouched	2	6	3			11
Bladelet	Microperforator		2				2
	Unretouched	4	2	1			7
Core on block		1					1
Total		19	81	63	9	5	177
