# Supplementary material for: Socio-economic dynamics of Magdalenian hunter-gatherers: Functional perspective
Source: PLoS One. 2022 Oct 5;17(10):e0274819. doi: 10.1371/journal.pone.0274819 (PMC9534454; doi:10.1371/journal.pone.0274819)
Supplement: S5 Table — Modified after Gauvrit Roux (2019). (PDF) [file pone.0274819.s006.pdf]

Level	Condition of the proximal edge of microliths	Fracture length (mm)								Total
		_	0-2	2-4	4-6	6-8	8-10	10-12	12-14	
**B2**	Bending fracture		1							1
**B3**	Burin-like fracture							1		1
	Bending fracture		1	2		1			2	6
	Bending fracture + burin-like and facial spin-off			1						1
	Snap fracture	10								10
	Snap fracture + burin-like and facial spin-off				1					1
	Snap fracture + facial spin-off		1	2		1				4
	Undetermined		2							2
	Intact	6								6
**B4**	Burin-like fracture		1	1	1				1	4
	Bending fracture		8	4						12
	Bending fracture + burin-like spin-off		1	1						2
	Bending fracture + facial spin-off			1						1
	Snap fracture	13								13
	Snap fracture + burin-like spin-off		1	2	1					4
	Snap fracture + burin-like and facial spin-off						1			1
	Snap fracture + facial spin-off		3	1						4
	Crushing	2								2
	Scars	3								3
	Scars + crushing	1								1
	Undetermined	3								3
	Intact	11								11
**B4+B5**	Bending fracture				1					1
	Bending fracture + facial spin-off					1				1
	Undetermined	1								1
**B5**	Burin-like fracture		1	1		1				3
	Burin-like fracture + facial spin-off						1			1
	Bending fracture		6	6	1			1		14
	Bending fracture + burin-like spin-off		1							1
	Bending fracture + burin-like and facial spin-off			1						1
	Bending fracture + facial spin-off		2		1					3
	Snap fracture	20								20
	Snap fracture + burin-like spin-off		1	1	1	1				4
	Snap fracture + burin-like and facial spin-off			1						1
	Snap fracture + facial spin-off		2	4						6
	Cone fracture	1								1
	Scars	2								2
	Undetermined	2								2
	Intact	13								13
**B6**	Bending fracture		2							2
	Snap fracture	2								2
**Undetermined**	Snap fracture	1								1
**Total**		91	34	29	7	5	2	2	3	173
