# Supplementary material for: Socio-economic dynamics of Magdalenian hunter-gatherers: Functional perspective
Source: PLoS One. 2022 Oct 5;17(10):e0274819. doi: 10.1371/journal.pone.0274819 (PMC9534454; doi:10.1371/journal.pone.0274819)
Supplement: S6 Table — Modified after Gauvrit Roux (2019). (PDF) [file pone.0274819.s007.pdf]

Level	Condition of the distal edge of microliths	Fracture length (mm)									Total
		_	0-2	2-4	4-6	6-8	8-10	10-12	12-14	16-24	
**B2**	Snap fracture + burin-like spin-off				1						1
**B3**	Burin-like fracture			1	1			1			3
	Bending fracture		4			1					5
	Bending fracture + burin-like spin-off				1		1				2
	Snap fracture	13									13
	Snap fracture + burin-like and facial spin-off					1					1
	Snap fracture + facial spin-off		1	1							2
	Intact	5									5
**B4**	Burin-like fracture		1	1							2
	Burin-like fracture + snap fracture			1							1
	Bending fracture		6	2	1						9
	Bending fracture + burin-like spin-off		1		2					1	4
	Bending fracture + facial spin-off		2	2							4
	Snap fracture	21									21
	Snap fracture + burin-like spin-off		2	3	1	1	1				8
	Snap fracture + facial spin-off		1	1	2						4
	Undetermined	1									1
	Intact	7									7
**B4+B5**	Bending fracture		1								1
	Snap fracture	1									1
	Snap fracture + burin-like spin-off						1				1
**B5**	Burin-like fracture		1	1	2	1				2	7
	Burin-like fracture + facial spin-off				1						1
	Bending fracture		7	3	1	1					12
	Bending fracture + burin-like spin-off				1						1
	Bending fracture + snap fracture						1				1
	Bending fracture + burin-like and facial spin-off		1	1					1		3
	Bending fracture + facial spin-off			2							2
	Snap fracture	19									19
	Snap fracture + burin-like spin-off		1	2						1	4
	Snap fracture + burin-like and facial spin-off						1				1
	Snap fracture + facial spin-off		5	2							7
	Intact	14									14
**B6**	Burin-like fracture			1		1					2
	Snap fracture + burin-like spin-off							1			1
	Intact	1									1
**Undetermined**	Snap fracture + burin-like spin-off				1						1
**Total**		82	34	24	15	6	5	2	1	4	173
