# Supplementary material for: Socio-economic dynamics of Magdalenian hunter-gatherers: Functional perspective
Source: PLoS One. 2022 Oct 5;17(10):e0274819. doi: 10.1371/journal.pone.0274819 (PMC9534454; doi:10.1371/journal.pone.0274819)
Supplement: S7 Table — Modified after Gauvrit Roux (2019). (PDF) [file pone.0274819.s008.pdf]

| Condition of the proximal edge of microliths | Fracture length (mm) | | | | | | | Total |
|---|---|---|---|---|---|---|---|---|
| | _ | 0-2 | 2-4 | 4-6 | 6-8 | 8-10 | 10-12 | |
| Burin-like fracture | | | 2 | 2 | 1 | 2 | 2 | 9 |
| Burin-like fracture + facial spin-off | | | | | 1 | | | 1 |
| Fracture en flexion | | 31 | 15 | 4 | 2 | | | 52 |
| Fracture en flexion + burin-like fracture | | | | | | 1 | | 1 |
| Snap fracture | 66 | | | | | | | 66 |
| Snap fracture + burin-like spin-off | | | 2 | | | | | 2 |
| Snap fracture + facial spin-off | | 3 | | | | | | 3 |
| Scars | 4 | | | | | | | 4 |
| Scars + crushing | 1 | | | | | | | 1 |
| Undetermined | 6 | | | | | | | 6 |
| Intact | 38 | | | | | | | 38 |
| Total | 115 | 34 | 19 | 6 | 4 | 3 | 2 | 183 |
