# Supplementary material for: Socio-economic dynamics of Magdalenian hunter-gatherers: Functional perspective
Source: PLoS One. 2022 Oct 5;17(10):e0274819. doi: 10.1371/journal.pone.0274819 (PMC9534454; doi:10.1371/journal.pone.0274819)
Supplement: S8 Table — Modified after Gauvrit Roux (2019). (PDF) [file pone.0274819.s009.pdf]

**Condition of the distal edge of microliths**	**Fracture length (mm)**									**Total**
	**_**	**0-2**	**2-4**	**4-6**	**6-8**	**8-10**	**10-12**	**12-14**	**16-18**	
Burin-like fracture		1	7		1		3	1	1	14
Bending fracture		18	9	6		2				35
Bending fracture + snap fracture				1						1
Snap fracture	98									98
Snap fracture + facial spin-off			1							1
Scars	4									4
Scars + crushing	1									1
Intact	29									29
Total	132	19	17	7	1	2	3	1	1	183
